# Safety and efficacy of programmed cell death-1 inhibitors in relapsed immune-privileged site lymphoma: A systematic review and meta-analysis

**DOI:** 10.1371/journal.pone.0319714

**Published:** 2025-04-29

**Authors:** Ekdanai Uawithya, Kamolchanok Kulchutisin, Jiraporn Jitprapaikulsan, Nattawut Leelakanok, Weerapat Owattanapanich

**Affiliations:** 1 Faculty of Medicine Siriraj Hospital, Mahidol University, Bangkok, Thailand; 2 Department of Transfusion Medicine, Faculty of Medicine Siriraj Hospital, Mahidol University, Bangkok, Thailand; 3 Division of Neurology, Department of Medicine, Faculty of Medicine Siriraj Hospital, Mahidol University, Bangkok, Thailand; 4 Department of Medicine, Division of Neurology, Faculty of Medicine Siriraj Hospital, Mahidol University, Siriraj Neuroimmunology Center, Bangkok, Thailand; 5 Division of Clinical Pharmacy, Faculty of Pharmaceutical Sciences, Burapha University, Chonburi, Thailand; 6 Division of Hematology, Department of Medicine, Faculty of Medicine Siriraj Hospital, Mahidol University, Bangkok, Thailand; 7 Faculty of Medicine Siriraj Hospital, Center of Excellence of Siriraj Adult Acute Myeloid/Lymphoblastic Leukemia, Mahidol University, Bangkok, Thailand; West China Hospital of Sichuan University, CHINA

## Abstract

**Background:**

Large B-cell lymphoma of immune-privileged sites (LBCL-IP) is a rare subtype characterized by immune evasion properties. Primary central nervous system lymphoma (PCNSL) and primary testicular lymphoma (PTL) are examples of LBCL-IP associated with programmed cell death protein 1 (PD-1). Few studies have investigated the use of PD-1 inhibitors in patients with relapsed PCNSL and PTL.

**Objective:**

To conduct a systematic review evaluating the efficacy and safety of PD-1 inhibitors in patients with relapsed PCNSL and PTL.

**Methods:**

We searched the PubMed, Embase, and Scopus databases for relevant studies. The inclusion criteria focused on adult patients diagnosed with relapsed PCNSL or PTL who were treated with PD-1 inhibitors. We excluded case reports or series with fewer than five participants, review articles, and animal studies. A random-effects model with the DerSimonian‒Laird method analyzed the pooled complete response rate (CRR), partial response rate (PRR), overall response rate (ORR), and progression-free survival (PFS) rate.

**Results:**

Seven studies comprising 127 patients (124 with relapsed PCNSL and 3 with PTL) were included. All patients were treated with either nivolumab or pembrolizumab. The pooled CRR was 42.8% (95% CI, 25.7%‒60.0%; I^2^ = 75.25%; p < 0.001), indicating high heterogeneity. The pooled PRR was 17.1% (95% CI, 9.5%‒24.7%; I^2^ = 18.71%; p = 0.287), with nonsignificant heterogeneity. The pooled ORR was 67.1% (95% CI, 44.9%‒89.4%; I^2^ = 88.64%; p < 0.001), indicating high heterogeneity. The 6-month PFS rate was 34.8% (95% CI, 18.1%‒51.5%; I^2^ = 27%; p = 0.242), with low heterogeneity. Thirty-eight adverse events were reported. The most common were skin reactions (14 events; 36.8%), fatigue (11 events; 28.9%), and nausea (6 events; 15.8%).

**Conclusions:**

Our study demonstrates that PD-1 inhibitors show promising efficacy in relapsed PCNSL and PTL, with significant responses observed. The adverse effects were mild, with the most common being skin reactions. Therefore, PD1 inhibitors have the potential to drive advancements in treatment strategies for relapsed PCNSL and PTL.

## Introduction

Large B-cell lymphoma of immune-privileged sites (LBCL-IP) is a rare lymphoma originating from immune-privileged organs. Mutations in the *MYD88* and *CD79B* genes [1], which play crucial roles in the tumor’s biological framework, are linked to immune privilege and tumor immune evasion. Gain-of-function mutations in *CD79B* often coexist with *MYD88* mutations in extranodal lymphomas, such as primary central nervous system lymphoma (PCNSL) and primary testicular lymphoma (PTL). These co-occurring mutations contribute to lymphomagenesis and tumor progression.

According to Cancer 2020, *CD79B* mutations were present in 53% of PCNSL cases, while *MYD88* mutations were observed in 60%‒79% of cases; 20% of patients exhibited both mutations. In PTL, *CD79B* mutations were found in 43.4% of cases, *MYD88* mutations in 71%‒77%, and coexisting mutations in 35% of patients.

PCNSL is a rare type of non-Hodgkin’s lymphoma, accounting for only 4% of intracranial neoplasms [2]. It often presents with focal neurological deficits, altered mental status, and seizures. Due to its aggressive nature, the estimated survival rate without treatment is 1.5 months [3]. However, recent advancements in treatment, particularly methotrexate-based regimens used as first-line therapy, have significantly improved outcomes [4]. The 5-year overall survival rate has increased to 26%. Despite these advancements, relapses occur in up to 50% of PCNSL patients who receive adequate primary treatment [5].

The challenging nature of relapsed PCNSL necessitates a variety of treatment modalities. Traditionally, radiation therapy was the sole treatment option, but strategies have evolved [3]. According to the latest National Comprehensive Cancer Network guidelines, autologous stem cell transplantation is the primary approach for second-line treatment. Additionally, a combination of lenalidomide and ibrutinib is now considered equally important [6].

PTL is a rare and aggressive form of extranodal non-Hodgkin lymphoma that predominantly affects men over the age of 60 and is associated with a poor prognosis. PTL typically presents as a painless, firm testicular mass or hydrocele, often without constitutional symptoms at the time of diagnosis. Standard treatment generally combines chemotherapy—most notably the CHOP regimen (cyclophosphamide, doxorubicin, vincristine, and prednisone)—with rituximab, central nervous system prophylaxis, testicular radiation therapy, and orchidectomy [7,8].

Recent advancements in understanding the molecular characteristics of PTL have paved the way for emerging targeted therapies [9,10]. A pivotal protein for targeted therapy is programmed cell death protein 1 (PD-1), a checkpoint protein located on T-cell surfaces. PD-1 interacts with programmed cell death ligand 1 (PD-L1), often expressed on tumor cells, leading to the evasion of tumor cell death and disease progression [11].

Previous research has demonstrated varying levels of association between PD-L1 expression and relapses in PCNSL and PTL [1]. For instance, Hoang-Xuan et al. [19] observed a complete response rate of only 16%, whereas Nayak et al. [20] reported a significantly higher rate of up to 75% with PD-1 inhibitors. However, evidence supporting the use of PD-1 inhibitors in relapsed PCNSL and PTL patients remains limited, and their role as salvage treatment remains controversial. Therefore, we conducted this meta-analysis to evaluate the efficacy and safety of PD-1 inhibitors in these patient populations.

## Methods

### Search strategy and selection criteria

We registered this study’s protocol in the International Prospective Register of Systematic Reviews (PROSPERO; CRD42024523297). The inclusion criteria encompassed patients aged 18 years or older who were diagnosed with relapsed PCNSL or PTL and treated with PD-1 or PD-L1 inhibitors. Eligible studies also provided sufficient data on baseline characteristics and desired outcomes. The exclusion criteria included case reports or series with fewer than five participants, review articles, and animal studies.

Two independent researchers (E.U. and K.K.) conducted a comprehensive search of the Medline, Embase, and Scopus databases from inception to January 2024. They screened titles and abstracts for studies related to “lymphoma,” “central nervous system,” and “PD-1/PD-L1 inhibitors” using Medical Subject Headings without restrictions on language or ethnicity. Additionally, the investigators performed manual searches of bibliographies of relevant articles. Duplicate studies were removed prior to abstract screening by the two researchers. Any discrepancies were resolved through discussion, with final decisions made in consultation with a third author (W.O.). Details of the search terms are provided in S1 Table. This meta-analysis was conducted per the guidelines of the Preferred Reporting Items for Systematic Reviews and Meta-Analyses (PRISMA) [12].

### Data extraction and quality assessment

Two investigators (E.U. and K.K.) independently extracted data using a standardized data collection form. A third author (W.O.) reviewed and resolved any discrepancies, with final decisions reached through mutual consensus. The extracted data comprised publication details, sample size, patient demographics (age and sex), treatment specifics, complete response rate (CRR), partial response rate (PRR), overall response rate (ORR), progression-free survival (PFS) rate, overall survival (OS) rate, and drug toxicities.

The same two investigators also independently assessed the risk of bias in each study using the Newcastle‒Ottawa Scale. Discrepancies in their assessments were resolved through consensus discussions with a third author (J.J.). Following the Newcastle‒Ottawa Scale guidelines, studies were evaluated across three domains: selection, comparability, and outcomes [13]. Additionally, the ROBINS-I tool and the GRADE approach were utilized to assess the risk of bias and the quality of evidence across the included studies. Detailed assessments are provided in S1 Fig and S2 Table.

### Outcomes of the meta-analyses

The primary outcomes of this study were the pooled CRR, PRR, ORR, and PFS rate in patients with relapsed PCNSL and PTL treated with anti-PD-1 drugs as salvage therapy. The secondary outcome was the aggregated incidence of drug toxicities reported across all studies.

### Statistical analysis

We conducted meta-analyses of the pooled response rates to PD-1 inhibitor treatments. A binary random-effects model with the DerSimonian‒Laird method was employed and implemented via Open Meta-Analyst for Windows 8 [14]. We calculated the pooled prevalence of the outcomes of interest and their 95% confidence intervals (CIs) from the effect sizes obtained in a single-arm meta-analysis. A random-effects model was chosen due to the potential for high heterogeneity among the included studies.

Statistical heterogeneity was assessed using Cochran’s Q test and the I^2^ statistic. We categorized I^2^ values as follows: insignificant heterogeneity (< 25%), low heterogeneity (26%‒50%), moderate heterogeneity (51%‒75%), and high heterogeneity (> 75%). To explore potential sources of heterogeneity, we conducted subgroup analyses based on treatment types and disease characteristics. Due to the limited number of studies included, a funnel plot was not constructed for assessing publication bias [15]. A p-value less than 0.05 was considered statistically significant.

## Results

**Fig 1:**
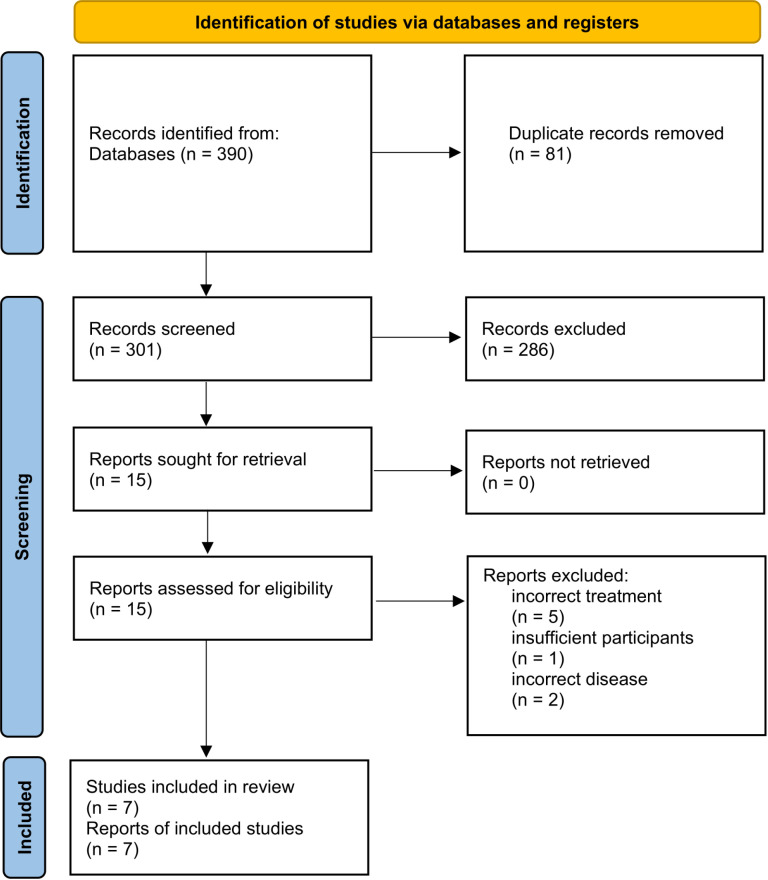
PRISMA 2020 flow diagram of the identified and included studies.

Through comprehensive searches in Medline, Scopus, and Embase, we identified 390 studies, including unpublished articles and abstracts. After removing 89 duplicates, 301 studies remained for title and abstract screening. From these, 15 studies were selected for full-text review. Ultimately, seven studies met the inclusion criteria and were included in our meta-analysis [16–22] (Fig 1). Additionally, we reported a case study with relevant content that did not fulfill the inclusion criteria in the results section [23].

### Baseline characteristics and quality assessment

A total of 127 patients were analyzed, comprising 124 diagnosed with relapsed PCNSL and 3 with PTL involving the central nervous system. The female-to-male ratio was 1.64:1, with 62.0% of patients being female. The median age was 65.5 years (range, 28–88 years). Nivolumab was administered to 52.8% of participants, while pembrolizumab was given to 47.2%. Notably, 60.6% of patients had previously received high-dose methotrexate-based chemotherapy.

The Newcastle–Ottawa Scale scores for the included studies ranged from 5 to 6 out of a maximum possible score of 8. All studies had a single treatment arm and were not evaluable for the comparability domain. Baseline characteristics, treatment regimens, and quality assessments of the included studies are summarized in **Table 1**.

**Table 1:** Baseline characteristics, treatment regimens, and quality assessments of the included studies.

References	Patients, n	Sex (M/F)	Age at onset, median (range)	Disease (PCNSL/PTL)	Treatment	Dose	Follow up time (months), median (range)	Cycle	Prior lines treatment before PD1 inhibitor	Newcastle–Ottawa scale
Nayak et al., 2017	5	NA	64 (54-85)	4/1	Nivolumab	3 mg/kg IV every 2 weeks	NA	NA	High-dose methotrexate, pemetrexed, high dose cytarabine	Selection:3 Comparability: 0 Outcome:3
Gavrilenko et al., 2020	8	2/6	62 (28-66)	7/1	Nivolumab	100 mg every 2 weeks	18 (3-44)	NA	High-dose methotrexate-based chemotherapy (1 patient)	Selection:3 Comparability: 0 Outcome:3
Hoang-Xuan et al., 2020	50	NA	72 (43-83)	50/0	Pembrolizumab	200 mg IV day of every 21 days, up to 2 years	6.7 (0.2-27.4)	NA	High-dose methotrexate-based chemotherapy	Selection:3 Comparability: 0 Outcome:3
Westin et al., 2023	18	4/14	63 (42-88)	18/0	Nivolumab	240 mg IV every 14 days, up to 2 years	NA	6 cycles	Ibrutinib followed by nivolumab or Ibrutinib combined with nivolumab	Selection:3 Comparability: 0 Outcome:3
Chuk wueke et al., 2023	10	5/5	74 (38-82)	10/0	Pembrolizumab	200 mg IV every 3 weeks	NA	NA	NA	Selection:2 Comparability: 0 Outcome:3
Gavrilenko et al., 2023	14	NA	NA	13/1	Nivolumab	NA	32	NA	NA	Selection:3 Comparability: 0 Outcome:3
Ho Yi et al., 2023	22	11/11	67 (37-82)	22/0	Nivolumab	3 mg/kg every 2 weeks	22.3 (13.1-31.5)	NA	High dose methotrexate procarbazine, vincristine (21 patients)	Selection:3 Comparability: 0 Outcome:3

**Abbreviations:** IV, intravenous; NA, not available; PCNSL, primary central nervous system lymphoma; PTL, primary testicular lymphoma; PD1, programmed cell death protein 1

### Treatment response and clinical outcomes

**Fig 2:**
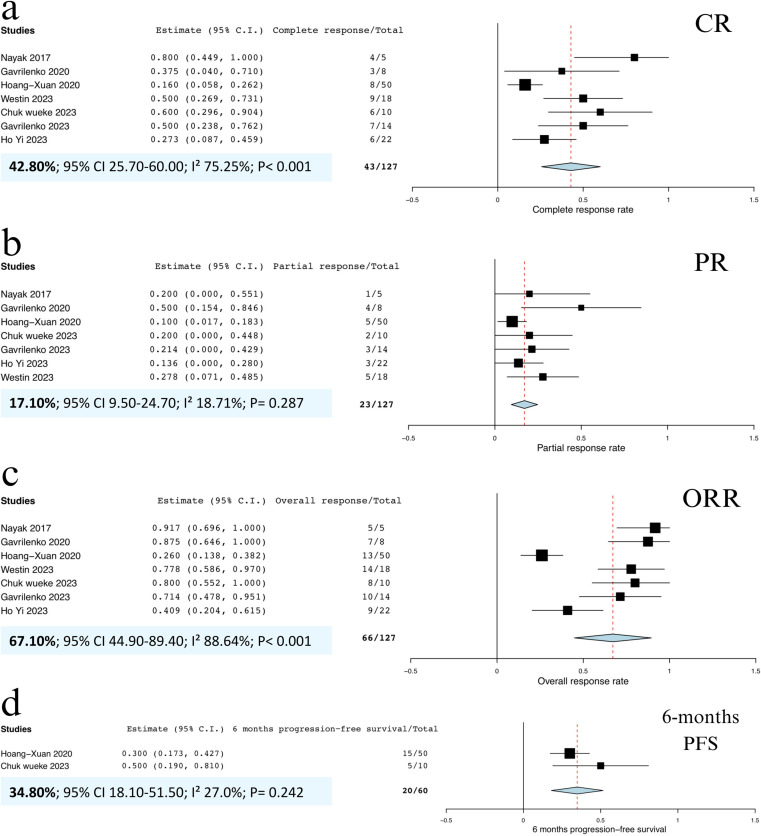
Forest plots of included studies demonstrating (a) pooled CRR of all PTL and PCNSL patients, (b) pooled PRR of all PTL and PCNSL patients, (c) pooled ORR of all PTL and PCNSL patients, and **(d)** 6-month PFS rate of all PTL and PCNSL patients. **Abbreviations:** CRR, complete response rate; ORR, overall response rate; PFS, progression-free survival; PRR, partial response rate.

All patients were evaluated for CR, PR, OR, and 6-month PFS. The pooled CRR was 42.8% (95% CI, 25.7%‒60.0%; I^2^ = 75.25%; p < 0.001; **Fig 2a**), indicating high heterogeneity. The pooled PRR was 17.1% (95% CI, 9.5%‒24.7%; I^2^ = 18.71%; p = 0.287; **Fig 2b**), with low heterogeneity. Additionally, the pooled ORR was 67.1% (95% CI, 44.9%‒89.4%; I^2^ = 88.64%; p < 0.001; **Fig 2c**), demonstrating high heterogeneity. Due to variability in follow-up times across studies, the pooled 6-month PFS rate was 34.8% (95% CI, 18.1%‒51.5%; I^2^ = 27.0%; p = 0.242; **Fig 2d**), reflecting low heterogeneity.

### Subgroup analysis of treatment response and clinical outcomes in relapsed PCNSL

**Fig 3:**
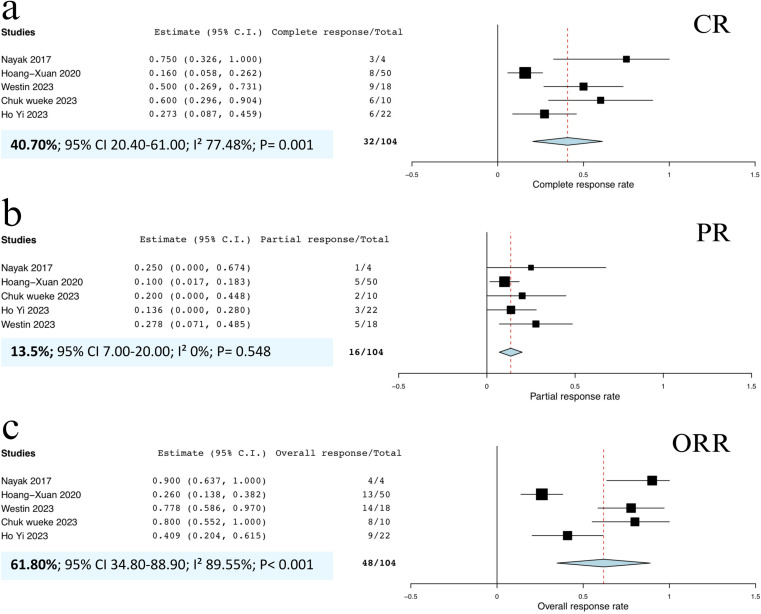
Forest plots of included studies demonstrating (a) pooled CRR of PCNSL patients, (b) pooled PRR of PCNSL patients, and (c) pooled ORR of PCNSL patients. **Abbreviations:** CRR, complete response rate; ORR, overall response rate; PRR, partial response rate.

Five studies [16,19–22] reported CRRs and ORRs for patients with relapsed PCNSL. Similarly, four studies [16,19,20,22] provided PRRs for this patient population. The pooled analysis demonstrated a CRR of 40.7% (95% CI, 20.4%‒61.0%; I^2^ = 77.48%; p = 0.001; **Fig 3a**) and a PRR of 13.5% (95% CI, 7.0%‒20.0%; I^2^ = 0%; p = 0.548; **Fig 3b**). Further analysis yielded a pooled ORR of 61.8% (95% CI, 34.8%‒88.9%; I^2^ = 89.55%; p < 0.001; **Fig 3c**).

### Subgroup analysis by PD1 inhibitor

**Fig 4:**
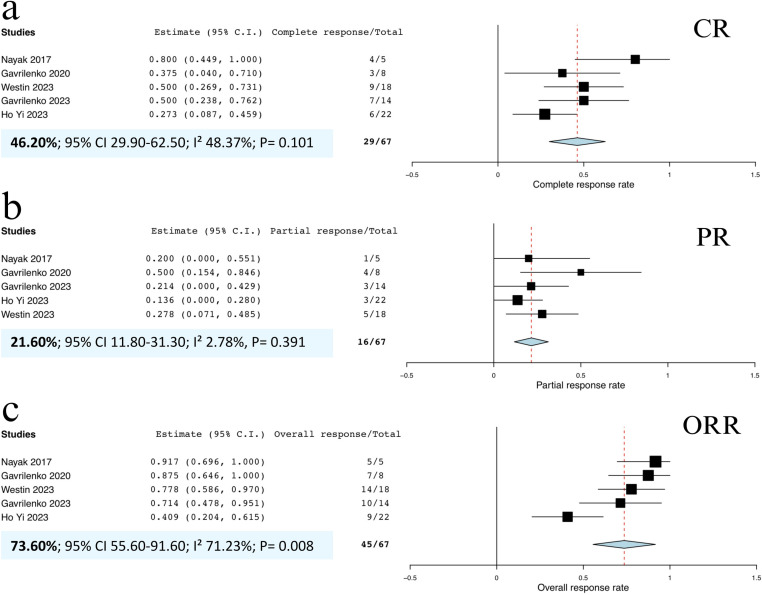
Forest plots of included studies demonstrating (a) pooled CRR of patients who received nivolumab, (b) pooled PRR of patients who received nivolumab, and (c) pooled ORR of patients who received nivolumab. **Abbreviations:** CRR, complete response rate; ORR, overall response rate; PRR, partial response rate.

We conducted subgroup analyses based on the type of PD-1 inhibitor administered. Despite the stratification, heterogeneity in the meta-analyses for the CRRs and ORRs remained substantial (I^2^ > 40%). For patients treated with nivolumab, the pooled CRR was 46.2% (95% CI, 29.9%‒62.5%; I^2^ **=** 48.37%; p **=** 0.101; **Fig 4a**), and the pooled PRR was 21.6% (95% CI, 11.8%‒31.3%; I^2^ = 2.78%; p = 0.391; **Fig 4b**). The pooled ORR for nivolumab was 73.6% (95% CI, 55.6%–91.6%; I^2^ = 71.23%; p = 0.008; **Fig 4c**).

**Fig 5:**
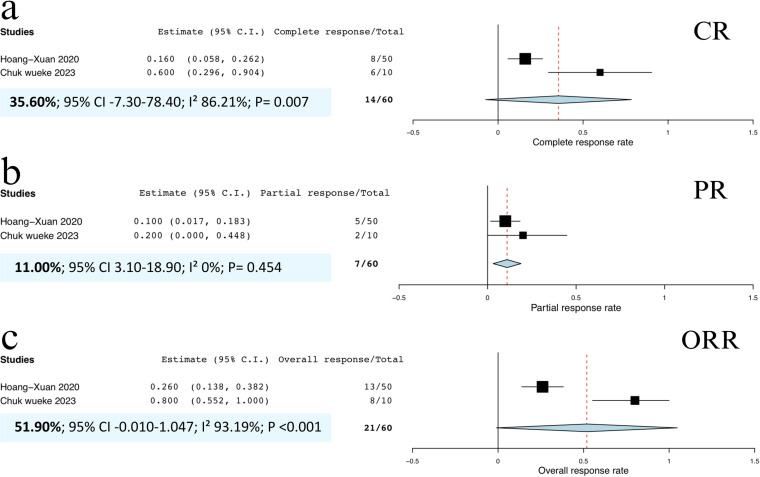
Forest plots of included studies demonstrating (a) pooled CRR of patients who received pembrolizumab, (b) pooled PRR of patients who received pembrolizumab, and (c) pooled ORR rate of patients who received pembrolizumab. **Abbreviations:** CRR, complete response rate; ORR, overall response rate; PRR, partial response rate.

In contrast, patients receiving pembrolizumab exhibited a pooled CRR of 35.6% (95% CI, –7.3% to 78.4%; I^2^ = 86.21%; p = 0.007; **Fig 5a**) and a pooled PRR of 11.0% (95% CI, 3.1%‒18.9%; I^2^ = 0%; p = 0.454; **Fig 5b**). The pooled ORR for pembrolizumab was 51.9% (95% CI, –0.01% to 104.7%; I^2^ = 93.19%; p < 0.001; **Fig 5c**). No statistically significant differences between the subgroups were detected. This conclusion is supported by the fact that the 95% confidence intervals for all outcomes in nivolumab users encompass the point estimates of the outcomes in pembrolizumab users [24]. Therefore, the efficacy of nivolumab and pembrolizumab appears comparable in the treatment of relapsed PCNSL.

### Treatment toxicities

Five studies [16,17,19–21] reported treatment toxicities associated with nivolumab and pembrolizumab. A total of 38 adverse drug reaction events were documented. The most common adverse effects were skin reactions, occurring in 14 cases (36.8%), and fatigue, reported in 11 cases (28.9%). Nausea was observed in six cases (15.8%) and mucositis in five cases (13.2%). Less frequently reported toxicities included elevated alanine aminotransferase/aspartate aminotransferase levels in one case (2.6%) and decreased renal function in one case (2.6%). Additional reports mentioned elevated liver function levels and flu-like symptoms; however, specific patient numbers were not provided.

### Excluded case reports

We identified one study reporting the outcome of a single patient with relapsed PCNSL treated with pembrolizumab 200 mg as salvage therapy [23]. The patient achieved complete remission at 20 months but subsequently developed inflammatory pneumonitis, which resolved with corticosteroid therapy.

## Discussion

This systematic review and meta-analysis is the first to evaluate the efficacy and safety of anti-PD-1 therapies in patients with relapsed PCNSL and PTL. Our findings indicate that anti-PD-1 inhibitors are promising emerging therapeutic options for these conditions. Specifically, we observed an overall CRR of 42.8%, a PRR of 17.1%, an ORR of 67.1%, and a 6-month PFS rate of 34.8%. Common adverse effects reported included skin reactions, fatigue, and nausea.

Historically, patients with relapsed PCNSL who did not receive further intervention had a median survival time of 14 months [25]. Whole-brain radiotherapy has been a standard salvage therapy, achieving ORRs of 74% to 79% and OS extending beyond 10 months [26, 27]. However, whole-brain radiotherapy is associated with both acute and long-term toxicities, such as cerebral edema, cognitive impairments, gait instability, and Parkinsonism-like symptoms. In recent years, ibrutinib—a tyrosine kinase inhibitor—has emerged as a promising alternative salvage treatment, demonstrating favorable response and survival rates [28–30]. Other salvage regimens that combine agents such as rituximab, temozolomide, and chemotherapy have shown variable efficacy, with ORRs ranging from 31% to 54% [31].

In lymphomas arising in immune-privileged sites, such as PCNSL and PTL, the tumor microenvironment is characterized by selective amplification of the 9p24.1 locus. This amplification leads to increased expression of PD-L1, PD-L2, and JAK2, enhancing the sensitivity of these lymphomas to immune checkpoint inhibitors targeting the PD-1/PD-L1 pathway, thereby offering significant therapeutic potential. However, these tumors also exhibit immune evasion mechanisms that may limit the effectiveness of PD-1/PD-L1 inhibitors. For example, the loss of MHC class I and II expression hinders T-cell recognition and correlates with poorer survival outcomes [32, 33]. Resistance to PD-1/PD-L1 inhibitors is further linked to mutations in the JAK-STAT pathway and reduced PD-L1 upregulation. Additionally, components of the tumor microenvironment—such as regulatory T cells (Tregs), myeloid-derived suppressor cells (MDSCs), and elevated cytokine levels like TGF-β—contribute to therapeutic resistance [34].

Prior treatments can significantly influence a patient’s prognosis when receiving anti-PD-1 inhibitors. For instance, whole-brain radiotherapy can enhance overall survival as a first-line therapy and may improve tumor response when combined with PD-1 inhibitors [35]. Although evidence suggests that combining whole-brain radiotherapy with anti-PD-1 therapies is effective in various cancers [36], no direct comparisons exist regarding the effects of whole-brain radiotherapy versus chemotherapy on the efficacy of PD-1 inhibitors as salvage therapy in PCNSL and PTL.

Comparative studies of nivolumab and pembrolizumab in patients with non-small cell lung cancer have shown no significant differences in the rates for CR, PR, PFS, and OS [37, 38]. However, notable disparities in efficacy between these agents have been observed in other malignancies. For instance, in hepatocellular carcinoma and metastatic melanoma, pembrolizumab has demonstrated a more substantial objective response than nivolumab [39, 40]. In contrast, our study suggests that nivolumab may yield a higher CRR, PRR, and ORR than pembrolizumab in the treatment of immune-privileged site lymphomas. Nevertheless, direct head-to-head comparisons are necessary to elucidate the differential efficacy of these drugs in this specific patient population.

Adverse effects commonly associated with anti-PD-1 inhibitors include dermatitis (17%), endocrine disorders (10%), pneumonitis (3%), hepatitis (3%), and colitis (2%), which align with our findings [41].

Despite these promising results, our study has several limitations. First, we did not perform a formal analysis for publication bias due to the limited number of studies and cases included. Nevertheless, publication bias is anticipated, especially since many abstracts were incorporated into this analysis. Systematic searches using only abstracts, titles, and keyword fields may miss over 50% of relevant studies [42]. This limitation suggests that important studies might have been overlooked if their abstracts did not contain keywords matching our search queries, even though their full texts are pertinent. Second, all included studies were small, and we employed a random-effects model, which may have skewed the pooled effects toward smaller studies [43]. Third, variations in prior treatments—such as the administration of methotrexate before anti-PD-1 inhibitors—could influence treatment responses and survival outcomes, potentially confounding our results.

Furthermore, the included studies exhibited variability in clinical baselines and treatment protocols. Some patients received PD-1 inhibitors as monotherapy, whereas others received them in combination with chemotherapy or other agents. This heterogeneity may affect the consistency of treatment responses and outcomes across studies. High heterogeneity was also observed in the CRR and ORR, necessitating caution when interpreting these findings. Additionally, the duration of follow-up varied among the studies, leading to inconsistencies in reporting long-term OS and PFS data.

Future research should focus on conducting randomized controlled trials to evaluate anti-PD-1 therapy in patients with relapsed refractory LBCL-IP, explicitly focusing on survival rates with adequate follow-up periods. Integrating biomarkers into treatment strategies may provide clinical benefits in managing LBCL-IP. Moreover, the potential role of anti-PD-1 combination therapies in newly diagnosed immune-privileged site lymphomas should be further explored.

## Conclusions

Our meta-analysis demonstrates the efficacy of anti-PD-1 inhibitors—specifically, nivolumab and pembrolizumab—in treating relapsed PCNSL and PTL. However, further research is needed to determine the most effective treatment protocols and to collect long-term follow-up data. This approach will enhance our understanding and application of PD-1 inhibitors in managing LBCL-IP.

## Supporting information

S1 Table Full details of search terms used in this study. (DOCX)

S1 Fig Overview of the Risk of Bias in Non-Randomized Studies of Interventions (ROBINS-I) Tool. (DOCX)

S2 Table GRADE Assessment of the Evidence on the Efficacy and Safety of Anti-PD-1 Therapies in Relapsed PCNSL and PTL. (DOCX)

S1 Dataset Comprehensive List of Included and Excluded Studies with Reasons. (DOCX)

S1 Checklist PRISMA 2020 Checklist. (DOCX)

S2 Dataset Collected data from included studies. (XLSX)
