# Supplementary material for: Safety and efficacy of programmed cell death-1 inhibitors in relapsed immune-privileged site lymphoma: A systematic review and meta-analysis
Source: PLoS One. 2025 Apr 29;20(4):e0319714. doi: 10.1371/journal.pone.0319714 (PMC12040093; doi:10.1371/journal.pone.0319714)
Supplement: S1 Table — (DOCX) [file pone.0319714.s001.docx]

**S1Table**: Full details of search terms used in this study

| Database | Search term | Number of studies retrieved |
| --- | --- | --- |
| Medline | (("Central Nervous System Neoplasms"[Mesh]) AND ("Programmed Cell Death 1 Receptor"[Mesh] OR "Nivolumab"[Mesh] OR "pembrolizumab" [Supplementary Concept] OR "avelumab" [Supplementary Concept] AND "atezolizumab" [Supplementary Concept] OR "cemiplimab" [Supplementary Concept] OR "durvalumab" [Supplementary Concept] OR "dostarlimab" [Supplementary Concept] OR "tislelizumab" [Supplementary Concept])) AND "Lymphoma"[Mesh] | 15 |
| Embase | lymphoma AND ('central nervous system'/exp OR 'central nervous system' OR 'testis tumor') AND ('pd-1'/exp OR 'pd-1' OR nivolumab OR pembrolizumab OR avelumab OR atezolizumab OR cemiplimab OR durvalumab OR dostarlimab OR tislelizumab) AND primary | 314 |
| SCOPUS | TITLE-ABS-KEY ( ( primary AND 'lymphoma' ) AND ( 'pd-1' OR nivolumab' OR 'pembrolizumab' OR avelumab OR atezolizumab OR cemiplimab OR durvalumab OR dostarlimab OR tislelizumab ) AND ( central AND nervous AND system OR testis ) ) | 61 |
