# Supplementary figures and images for: Safety and efficacy of programmed cell death-1 inhibitors in relapsed immune-privileged site lymphoma: A systematic review and meta-analysis
Source: PLoS One. 2025 Apr 29;20(4):e0319714. doi: 10.1371/journal.pone.0319714 (PMC12040093; doi:10.1371/journal.pone.0319714)

**S2 Figure**: Overview of the Risk of Bias in Non-Randomized Studies of Interventions (ROBINS-I) Tool


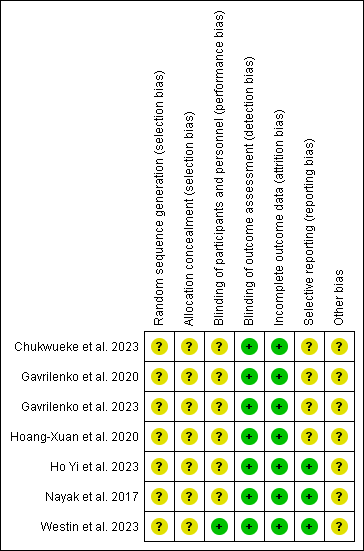

Supplement: S1 Fig — (DOCX) [file pone.0319714.s002.docx]
