# Supplementary material for: Safety and efficacy of programmed cell death-1 inhibitors in relapsed immune-privileged site lymphoma: A systematic review and meta-analysis
Source: PLoS One. 2025 Apr 29;20(4):e0319714. doi: 10.1371/journal.pone.0319714 (PMC12040093; doi:10.1371/journal.pone.0319714)
Supplement: S2 Table — (DOCX) [file pone.0319714.s003.docx]

**S3 Table:** GRADE Assessment of the Evidence on the Efficacy and Safety of Anti-PD-1 Therapies in Relapsed PCNSL and PTL

| **Study** | **Study design** | **Risk of bias** | **inconsistency** | **indirectness** | **Imprecision** | **Publication bias** | **Certainty** |
| --- | --- | --- | --- | --- | --- | --- | --- |
| Nayak 2017 | Observational study | Not serious | Not serious | Not serious | Serious | Not serious | 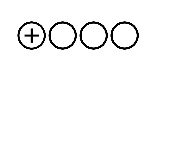Very low |
| Gavrilenko 2020 | Observational study | Not serious | Not serious | Not serious | Serious | Not serious | 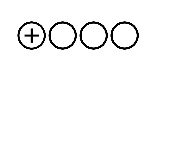Very low |
| Hoang-Xuan 2020 | Observational study | Not serious | Not serious | Not serious | Not serious | Not serious | 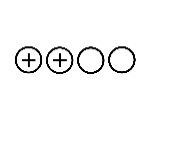Low |
| Westin 2023 | Observational study | Not serious | Not serious | Not serious | Not serious | Not serious | 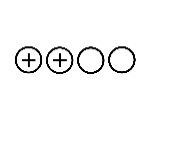Low |
| Chuk wueke 2023 | Observational study | Not serious | Not serious | Not serious | Not serious | Not serious | 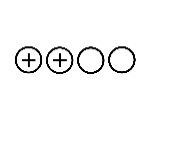Low |
| Gavrilenko 2023 | Observational study | Not serious | Not serious | Not serious | Not serious | Not serious | 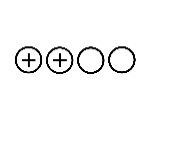Low |
| Ho Yi 2023 | Observational study | Not serious | Not serious | Not serious | Not serious | Not serious | 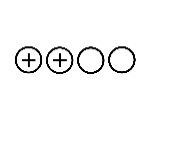Low |

GRADE indicates Grading of Recommendations, Assessment, Development, and Evaluation
