# Supplementary material for: Safety and efficacy of programmed cell death-1 inhibitors in relapsed immune-privileged site lymphoma: A systematic review and meta-analysis
Source: PLoS One. 2025 Apr 29;20(4):e0319714. doi: 10.1371/journal.pone.0319714 (PMC12040093; doi:10.1371/journal.pone.0319714)
Supplement: S1 Dataset — (DOCX) [file pone.0319714.s004.docx]

**S4 Dataset: Comprehensive List of Included and Excluded Studies with Reasons**

This dataset provides a comprehensive overview of all studies screened following duplication removal, including both included studies and excluded studies with the reasons for their exclusion.

**Excluded from title and abstract screening**

'13th International Conference on Malignant Lymphoma, 13 ICML', Hematol. Oncol., 33 (2015 2015).

A, LEVY, et al., Concurrent irradiation with the anti-programmed cell death ligand-1 immune checkpoint blocker durvalumab: Single centre subset analysis from a phase 1/2 trial (68; England, 2016) 156-62.

ABDULLA, M., et al., 'PD-L1 and IDO1 are potential targets for treatment in patients with primary diffuse large B-cell lymphoma of the CNS', Acta Oncol., 60/4 (2021 2021), 531-38.

ABELE, M., et al., 'Primary lung carcinoma in children and adolescents: An analysis of the European Cooperative Study Group on Paediatric Rare Tumours (EXPeRT)', Eur. J. Cancer, 175 (2022 2022), 19-30.

AKBULUT, D., et al., 'Immune checkpoint molecule (PD-1, PD-L1, PD-l2 ) expression, 9p24.1 gene locus alterations and tumor microenvironment status of primary central nervous system lymphoma and their clinical relevance: A quantitative analysis', Lab. Investig., 101 (2021 2021), 796-97.

ALIMONTI, P. AND GONZALEZ CASTRO, L.N., 'The Current Landscape of Immune Checkpoint Inhibitor Immunotherapy for Primary and Metastatic Brain Tumors', Antibodies, 12/2 (2023 2023).

ANAGNOSTOU, T. AND ANSELL, S.M., 'Immunomodulators in Lymphoma', Curr. Treat. Options Oncol., 21/4 (2020 2020).

ANDREA, M.L., et al., 'Rituximab treatment in B-cell NHL children in two Brazilian institutions', Br. J. Haematol., 171 (2015 2015), 81.

APOSTOLIDIS, J., et al., 'Current clinical applications and future perspectives of immune checkpoint inhibitors in non-Hodgkin lymphoma', J. Immunol. Res., 2020 (2020 2020).

ARDESHNA, K., et al., 'Study of AUTO3, the first bicistronic chimeric antigen receptor (CAR) targeting CD19 and CD22, followed by anti-PD1 consolidation in patients with relapsed/refractory (R/R) diffuse large B cell lymphoma (DLBCL): Alexander study', Blood, 132 (2018 2018).

ARDIZZONI, A., et al., 'Primary results from TAIL: A global single-arm safety study of atezolizumab monotherapy in a diverse population of patients with previously treated advanced non-small cell lung cancer', J. Immunother. Cancer, 9/3 (2021 2021).

AUTIER, L., et al., 'Cerebrospinal fluid interleukin-10 may be a useful biomarker for atypical primary central nervous system lymphoma relapse', Rev. Neurol., 177/4 (2021 2021), 436-39.

BARDEN, M.M. AND OMURO, A.M., 'Top advances of the year: Neuro-oncology', Cancer, 129/10 (2023 2023), 1467-72.

BARTA, S.K., et al., 'Pembrolizumab and Copanlisib for the Treatment of Relapsed or Refractory Mature T-Cell Lymphomas', Blood, 134 (2019 2019), 4031.

BATLEVI, C., et al., 'Distinctive genomic alterations in testicular diffuse large B cell lymphoma', Blood, 126/23 (2015 2015), 3655.

BELLUR, S., et al., 'Management of Brain Metastases: A Review of Novel Therapies', Semin. Neurol., 43/6 (2023 2023), 845-58.

BENEVOLO SAVELLI, C., et al., 'Chimeric Antigen Receptor-T Cell Therapy for Lymphoma: New Settings and Future Directions', Cancers, 16/1 (2024 2024).

BENNANI, N.N., et al., 'Copanlisib in Combination with Nivolumab in Subjects with Relapsed/Refractory Diffuse Large B-Cell Lymphoma and Primary Mediastinal Large B-Cell Lymphoma: A Phase 2 Study', Blood, 134 (2019 2019), 4090.

BERGHOFF, A.S., et al., 'Predictive molecular markers in metastases to the central nervous system: recent advances and future avenues', Acta Neuropathol., 128/6 (2014 2014), 879-91.

BOUMAZA, X., et al., 'Progressive Multifocal Leukoencephalopathy Treated by Immune Checkpoint Inhibitors', Ann. Neurol., 93/2 (2023 2023), 257-70.

BREZINA, T., et al., 'First-line high-dose therapy and autologous blood stem cell transplantation in patients with primary CNS non-hodgkin lymphomas', Bone Marrow Transplant., 53 (2019 2019), 609.

BS, GEURTS, et al., 'Efficacy, safety and biomarker analysis of durvalumab in patients with mismatch-repair deficient or microsatellite instability-high solid tumours', BMC cancer, 23/1 (2023-3-4 2023), 205.

C, BALDINI, et al., '[Not Available]', Bulletin du cancer, 105 (2018-12 2018), S59-S67.

CALIMERI, T., STEFFANONI, S., AND BATCHELOR, T.T., 'Innovative Therapeutic Strategies for Primary CNS Lymphoma', Curr. Treat. Options Neurol., 23/4 (2021 2021).

CARPIO, C., et al., 'Trial in Progress: Phase 1 Trial Evaluating the Safety and Tolerability of Odronextamab in Combination with Cemiplimab in Relapsed/Refractory Aggressive B-Cell Non-Hodgkin Lymphoma', Blood, 142 (2023 2023), 3100.

CASTEL, M., et al., 'Primary central nervous system lymphoma following immunotherapy for metastatic melanoma', Lymphome cérébral primitif après immunothérapie d'un mélanome métastatique, 146/10 (2019 2019), 634-39.

CHAMBERLAIN, M.C., et al., 'Systemic therapy of brain metastases: Non-small cell lung cancer, breast cancer, and melanoma', Neuro-Oncology, 19/1 (2017 2017), i1-i24.

CHAN, T.S.Y., et al., 'Low-dose nivolumab induced durable complete response in relapsed primary central nervous system diffuse large B cell lymphoma', Ann. Hematol., 98/9 (2019 2019), 2227-30.

CHAPUY, B., et al., 'Actionable genetic features of primary testicular and primary central nervous system lymphomas', Blood, 124/21 (2014 2014).

CHEN, J.P., 'Immunotherapy application for advanced cancers: One institution experiences since 2016 to 2019', Ann. Oncol., 30 (2019 2019), ix105.

CHEN, X., et al., 'PD-1 Blockade Combined with R-CHOP in Patients with Newly Diagnosed EBV-Positive Diffuse Large B-Cell Lymphoma: A Retrospective Analysis', Blood, 142 (2023 2023), 4506.

CHEN, X., et al., 'Toripalimab plus lenalidomide for central nervous system recurrence in refractory CD5+ diffuse large B-cell lymphoma with MYD88 and CD79B comutation: a case report', Transl. Cancer Res., 13/2 (2024 2024), 1188-95.

CHENG, C.-L., et al., 'Clinicopathologic analysis of programmed cell death 1 and its ligands PD-L1/PD-L2 expressions in primary central nervous system lymphoma', HemaSphere, 2 (2018 2018), 92.

CHERKASHINA, I.V., et al., 'Treatment of primary central nervous system lymphomas', Oncogematologiya, 16/2 (2021 2021), 10-20.

CHO, H., et al., 'Programmed cell death 1 expression is associated with inferior survival in patients with primary central nervous system lymphoma', Oncotarget, 8/50 (2017 2017), 87317-28.

CHO, I., et al., 'PD-L1 Expression in Patients with Primary Central Nervous System Diffuse Large B-Cell Lymphoma: Its Correlation with Serum Levels of Soluble Programmed Death-Ligand 1 (sPD-L1)', Blood, 134 (2019 2019), 1601.

CHO, I., et al., 'Serum levels of soluble programmed death-ligand 1 (sPD-L1) in patients with primary central nervous system diffuse large B-cell lymphoma', BMC Cancer, 20/1 (2020 2020).

CHRISTIAN, B., 'Checkpoint inhibition in CNS lymphoma', Blood, 129/23 (2017 2017), 3045-46.

CHUKWUEKE, U.N. AND NAYAK, L., 'Central Nervous System Lymphoma', Hematol. Oncol. Clin. North Am., 33/4 (2019 2019), 597-611.

CHUKWUEKE, U., et al., 'A PHASE 1B STUDY OF PEMBROLIZUMAB, IBRUTINIB AND RITUXIMAB IN RECURRENT/REFRACTORY (RR) PRIMARY CENTRAL NERVOUS SYSTEM LYMPHOMA (PCNSL)', Neuro-Oncology, 25 (2023 2023), v71-v72.

COHEN, Y.I., et al., 'Long-Term Results with Thiotepa-Containing Conditioning Regimens for Autologous Stem Cell Transplantation', Transplant. Cell. Ther, 29/8 (2023 2023), 505.e1-05.e8.

CRESPO, M., et al., 'Reversal of immune tolerance and increased anti tumoral immune response in a mouse model of CNS B cell lymphoma after combined XPO1 and BCR inhibition', Cancer Res., 78/13 (2018 2018).

CUNNINGHAM, J., IYENGAR, S., AND SHARMA, B., 'Evolution of lymphoma staging and response evaluation: Current limitations and future directions', Nat. Rev. Clin. Oncol., 14/10 (2017 2017), 631-45.

D, WEI, et al., 'Recognizing encephalopathy in immune checkpoint inhibitor therapy: A single-center experience', Cancer medicine, 10/9 (2021-5 2021), 2978-86.

D, POUESSEL, et al., Hypofractionated Stereotactic Re-irradiation and

Anti-PDL1 Durvalumab Combination in Recurrent Glioblastoma: STERIMGLI Phase I Results (28; England, 2023) 825-e17.

D, WANG, et al., 'Dynamics of tumor in situ fluid circulating tumor DNA in recurrent glioblastomas forecasts treatment efficacy of immune checkpoint blockade coupled with low-dose bevacizumab', Journal of cancer research and clinical oncology, 150/10 (2024-10-18 2024), 466.

DAGOGO-JACK, I., et al., 'Treatment of brain metastases in the modern genomic era', Pharmacol. Ther., 170 (2017 2017), 64-72.

DAVALOS, V. AND ESTELLER, M., 'Cancer epigenetics in clinical practice', CA Cancer J. Clin., 73/4 (2023 2023), 376-424.

DAVIS, K.L., et al., 'Nivolumab in children and young adults with relapsed or refractory solid tumours or lymphoma (ADVL1412): a multicentre, open-label, single-arm, phase 1–2 trial', Lancet Oncol., 21/4 (2020 2020), 541-50.

DE-LA-FUENTE, C., et al., 'Pembrolizumab for refractory primary mediastinal B-cell lymphoma with central nervous system involvement', Hematol. Oncol., 39/3 (2021 2021), 419-22.

DELATTRE, J.-Y., 'Improving diagnosis and management of primary brain tumors', Curr. Opin. Neurol., 30/6 (2017 2017), 639-42.

DESAI, A.V., et al., 'Updated entrectinib data from a phase 1/2 trial in children and adolescents with recurrent or refractory solid tumors, including primary central nervous system (CNS) tumors', Pediatr. Blood Cancer, 67 (2020 2020).

DI BLASI, R., et al., 'Outcome of relapsed/refractory aggressive b-cell lymphoma patients relapsing after anti-CD19 car t-cells and enrolled in the descar-t french national registry', Blood, 138 (2021 2021), 885.

DI CINTIO, F., et al., 'The Molecular and Microenvironmental Landscape of Glioblastomas: Implications for the Novel Treatment Choices', Front. Neurosci., 14 (2020 2020).

DZIADZIUSZKO, R., et al., 'Blood First Assay Screening Trial (BFAST) in Treatment-Naive Advanced or Metastatic NSCLC: Initial Results of the Phase 2 ALK-Positive Cohort', J. Thorac. Oncol., 16/12 (2021 2021), 2040-50.

EBRAHIMI, S., et al., 'Immune checkpoint inhibitors therapy as the game-changing approach for pediatric lymphoma: A brief landscape', Crit. Rev. Oncol. Hematol., 193 (2024 2024).

ECKBURG, A., JOHN, J., AND KUMTHEKAR, P., 'Investigating the impact of NGS data availability on clinical decision-making in brain cancer', J. Clin. Oncol., 40/16 (2022 2022).

EL-TAWAB, R., et al., 'Promising Effect of PDL1 Inhibitors in the Front-Line Management of Primary Aggressive Central Nervous System Lymphoma: A Case Report', Hematol. Oncolog. Stem Cell Ther., 16/2 (2023 2023), 151-53.

EUSKIRCHEN, P. AND PEYRE, M., 'Management of meningioma', Presse Med., 47/11 (2018 2018), e245-e52.

FALCHOOK, G.S., et al., 'A first-in-human phase I dose-escalation trial of the B7-H6/CD3 T-cell engager BI 765049 ± ezabenlimab (BI 754091) in patients with advanced solid tumors expressing B7-H6', J. Clin. Oncol., 40/16 (2022 2022).

FEI, F., WANG, K., AND PEKER, D., 'Characterization of cancer immune landscape in primary central nervous system lymphoma', Erciyes. med. J., 43/5 (2021 2021), 487-93.

FENG, L., et al., 'BTK inhibitor combined with anti-PD-1 monoclonal antibody for the treatment of CD20-negative primary central nervous system lymphoma: A case report', Oncol. Lett., 25/2 (2023 2023).

FERRERI, A.J.M., et al., 'Primary central nervous system lymphomas: EHA–ESMO Clinical Practice Guideline for diagnosis, treatment and follow-up☆', Ann. Oncol., 35/6 (2024 2024), 491-507.

FROSCH, Z.A., et al., 'Outcomes for Double Hit Lymphoma Patients Identified Via Routine Vs Selective Testing for MYC Rearrangement', Blood, 134 (2019 2019), 1607.

FURUSE, M., et al., 'Immunotherapy of nivolumab with dendritic cell vaccination is effective against intractable recurrent primary central nervous system lymphoma: A case report', Neurol. Med.-Chir., 57/4 (2017 2017), 191-97.

G, TAPIA RICO, et al., 'Metastatic myxopapillary ependymoma treated with immunotherapy achieving durable response', (13; England2020).

GANDHI, M.K., et al., 'The impact of EBV upon the tumor microenvironment and mutational profile of primary CNS lymphoma in PTLD', Blood, 130 (2017 2017).

GANDHI, M.K., et al., 'EBV+ CNS lymphomas have a distinctive tumor microenvironment and genetic profile, which is amenable to combination 3rd party EBV-specific CTL and ibrutinib therapy', Hematol. Oncol., 37 (2019 2019), 130-32.

GATSON, N.T., et al., 'Immuno-oncotherapy (IO) provoked encephalitis and metastatic mimicry: Patient with history of renal cell carcinoma on IO presents with seizure, field cut, and brain mass', Neuro-Oncology, 21 (2019 2019), vi182.

GEOERGER, B., et al., 'KEYNOTE-051: An update on the phase 2 results of pembrolizumab (pembro) in pediatric patients (pts) with advanced melanoma or a PD-L1-positive advanced, relapsed or refractory solid tumor or lymphoma', J. Clin. Oncol., 36/15 (2018 2018).

GHAFOURI, S., et al., 'Axicabtagene Ciloleucel CAR T-cell therapy for relapsed/refractory secondary CNS non-Hodgkin lymphoma: comparable outcomes and toxicities, but shorter remissions may warrant alternative consolidative strategies?', Bone Marrow Transplant., 56/4 (2021 2021), 974-77.

GONG, J., et al., 'Squamous cell transformation of primary lung adenocarcinoma in a patient with EML4-ALK fusion variant 5 refractory to ALK inhibitors', JNCCN J. Nat. Compr. Cancer Netw., 17/4 (2019 2019), 297-301.

GOTFRIT, J., et al., 'Determinants of the Cancer Drug Funding Process in Canada', Curr. Oncol., 29/3 (2022 2022), 1997-2007.

GRAHAM, M.S. AND DEANGELIS, L.M., 'Improving outcomes in primary CNS lymphoma', Best Pract. Res. Clin. Haematol., 31/3 (2018 2018), 262-69.

GROMMES, C., et al., 'Introduction of novel agents in the treatment of primary CNS lymphoma', Neuro-Oncology, 21/3 (2019 2019), 306-13.

GROMMES, C., 'Central Nervous System Lymphomas', CONTINUUM Lifelong Learn. Neurol., 26/6 (2020 2020), 1476-94.

H, PENG, X, HE, AND Q, WANG, 'Immune checkpoint blockades in gynecological cancers: A review of clinical trials', Acta obstetricia et gynecologica Scandinavica, 101/9 (2022-9 2022), 941-51.

H, HIKINO, et al., '[A Case of Breast Cancer Brain Metastases Successfully Treated with Pembrolizumab Therapy after Disease Progression with Atezolizumab Therapy]', (50; Japan2023), 1456-58.

HADDAD, R., et al., 'Solitary Primary Central Nervous System Lymphoma Mimicking Third Ventricular Colloid Cyst—Case Report and Review of Literature', World Neurosurg., 123 (2019 2019), 286-94.

HAN, C.H. AND BATCHELOR, T.T., 'Diagnosis and management of primary central nervous system lymphoma', Cancer, 123/22 (2017 2017), 4314-24.

HATIC, H., SAMPAT, D., AND GOYAL, G., 'Immune checkpoint inhibitors in lymphoma: challenges and opportunities', Ann. Transl. Med., 9/12 (2021 2021).

HAWKES, E.A., et al., 'AvR-CHOP: Feasibility Study of Induction and Maintenance Avelumab Plus R-CHOP in Patients with Diffuse Large B-Cell Lymphoma (DLBCL)', Blood, 134 (2019 2019), 5332.

HAYANO, A., et al., 'Programmed cell death ligand 1 expression in primary central nervous system lymphomas: A clinicopathological study', Anticancer Res., 37/10 (2017 2017), 5655-66.

HEPNER, A., et al., 'Serum neurofilament light, glial fibrillary acidic protein and tau are possible serum biomarkers for activity of brain metastases and gliomas', World J. Oncol., 10/4 (2019 2019), 169-75.

HERNÁNDEZ, P.N., et al., 'A rare gastrointestinal tumor: primary gastric melanoma', Rev. Esp. Enferm. Dig., 115/5 (2023 2023), 278-79.

HEß, G., 'Checkpoint Inhibition in Non-Hodgkin's Lymphoma', Oncol. Res. Treat., 40/11 (2017 2017), 662-72.

HIDA, T., et al., 'Atezolizumab in Japanese Patients With Previously Treated Advanced Non–Small-Cell Lung Cancer: A Subgroup Analysis of the Phase 3 OAK Study', Clin. Lung Cancer, 19/4 (2018 2018), e405-e15.

HILAL, T., 'Primary central nervous system lymphoma: Consensus, controversies, and future directions', Adv.Cell Gene Ther., 3/3 (2020 2020).

HOLDHOFF, M., WAGNER-JOHNSTON, N., AND ROSCHEWSKI, M., 'Systemic approach to recurrent primary cns lymphoma: Perspective on current and emerging treatment strategies', OncoTargets Ther., 13 (2020 2020), 8323-35.

HÖLLEIN, A., et al., 'Characteristics and outcomes of patients with cancer and COVID-19: results from a cohort study', Acta Oncol., 60/1 (2021 2021), 24-27.

HOUDA, I., et al., 'New systemic treatment paradigms in resectable non-small cell lung cancer and variations in patient access across Europe', Lancet. Reg. Health. Eur., 38 (2024 2024).

HU, Z., et al., 'Nivolumab and ipilimumab population pharmacokinetics in support of pediatric dose recommendations—Going beyond the body-size effect', CPT Pharmacometrics Syst. Pharmacol., 13/3 (2024 2024), 476-93.

HUTCHINGS, M., et al., 'ESMO consensus conference on Malignant lymphoma: Management of ‘ultra-high-risk’ patients', Ann. Oncol., 29/8 (2018 2018), 1687-700.

HUTCHINGS, M., et al., 'Glofitamab (Glofit) in Combination with Polatuzumab Vedotin (Pola): Phase Ib/II Preliminary Data Support Manageable Safety and Encouraging Efficacy in Relapsed/Refractory (R/R) Diffuse Large B-Cell Lymphoma (DLBCL)', Blood, 138 (2021 2021), 525.

ILLERHAUS, G., SCHORB, E., AND KASENDA, B., 'Novel agents for primary central nervous system lymphoma: Evidence and perspectives', Blood, 132/7 (2018 2018), 681-88.

ILLERHAUS, G., 'Targeted therapy in central nervous system lymphoma', Oncol. Res. Treat., 43 (2020 2020), 20-21.

INFANTE, J.R., et al., 'PEGylated human IL-10 (AM0010) in advanced solid tumors', Mol. Cancer Ther., 14/12 (2015 2015).

ISABELLE, C., et al., 'Therapeutic Potential and Role of CD38 in Cutaneous T-Cell Lymphoma Pathogenesis', Blood, 140 (2022 2022), 9216-18.

JACOBSON, C.A., 'Pilot Data on Axicabtagene Ciloleucel for CNS Lymphoma', Clin. Adv. Hematol. Oncol., 22/6 (2024 2024), 268-70.

JAEGER, U., et al., 'Portia: A phase 1b study evaluating safety and efficacy of tisagenlecleucel and pembrolizumab in patients with relapsed/refractory diffuse large B-cell lymphoma', Hematol. Oncol., 37 (2019 2019), 560.

JEUNG, H.-C., OH, S.E., AND KIM, J.H., 'Immune-related Adverse Events: Overview and Management Strategies for the Use of Immune Checkpoint Inhibitors', J. Rheum. Dis., 26/4 (2019 2019), 221-34.

JIA, L., et al., 'Refractory primary central nervous system lymphoma treated with programmed death⁃1 monoclonal antibody and Bruton tyrosine kinase inhibitor combined with rituximab: report of 1 case and review of literature', 程序性死亡受体 1 单抗及布鲁顿酪氨酸激酶抑制剂联合利妥昔单抗治疗难治原发中枢神经系统淋巴瘤 1 例并文献复习, 31/12 (2022 2022), 734-37.

JIMÉNEZ, I., et al., 'Repolarization of tumor infiltrating macrophages and increased survival in mouse primary CNS lymphomas after XPO1 and BTK inhibition', J. Neuro-Oncol., 149/1 (2020 2020), 13-25.

JIN, Q.-Q., et al., 'Whole Exome Sequencing Reveals Gene Mutation Characteristics of Primary Central Nervous System Lymphoma', Zhongguo Shi Yan Xue Ye Xue Za Zhi, 32/3 (2024 2024), 756-62.

JORDAN, B., et al., 'Successful use of an immune checkpoint inhibitor in a patient with myasthenia gravis in remission', Muscle Nerve, 60/1 (2019 2019), E7-E8.

JOSHI, M. AND ANSELL, S.M., 'Activating the Antitumor Immune Response in Non-Hodgkin Lymphoma Using Immune Checkpoint Inhibitors', J. Immunol. Res., 2020 (2020 2020).

KAHL, B.S., et al., 'The VcR-CVAD regimen produces a high complete response rate in untreated mantle cell lymphoma (MCL): First analysis of E1405 - A phase II study of VcR-CVAD with maintenance rituximab for MCL', Blood, 114/22 (2009 2009).

KAHL, B.S., et al., 'Mature results from ecog study E1405-a phase II study of VCR-CVAD with maintenance rituximab for previously untreated mantle cell lymphoma', Blood, 120/21 (2012 2012).

KALASAUSKAS, D., et al., 'Beyond Glioma: The Utility of Radiomic Analysis for Non-Glial Intracranial Tumors', Cancers, 14/3 (2022 2022).

KAMABU, L.K., et al., 'Primary malignant melanoma, an atypical presentation in the cervical spine: a case report', J. Med. Case Rep., 17/1 (2023 2023).

KARAMCHANDANI, J., et al., 'Primary T-cell lymphoma of the CNS expressing PD-1, a marker of germinal center T-cells', J. Neuropathol. Exp. Neurol., 68/5 (2009 2009), 589-90.

KARSCHNIA, P., et al., 'Car t‐cells for cns lymphoma: Driving into new terrain?', Cancers, 13/10 (2021 2021).

KASI, P.M., BLOCK, M.S., AND ANSELL, S.M., 'Treatment of HIV/AIDS associated cancers with immunotherapy targeting PD-1/PD-L1 instead of chemotherapy', Med. Hypotheses, 86 (2016 2016), 129-31.

KASSA, C., et al., 'Successful nivolumab therapy in an allogeneic stem cell transplant child with post-transplant lymphoproliferative disorder', Pediatr. Transplant., 22/8 (2018 2018).

KAULEN, L.D., et al., 'Intraventricular immune checkpoint inhibition with nivolumab in relapsed primary central nervous system lymphoma', NeuroOncol. Adv., 4/1 (2022 2022).

KAULEN, L.D. AND BAEHRING, J.M., 'Treatment Options for Recurrent Primary CNS Lymphoma', Curr. Treat. Options Oncol., 23/11 (2022 2022), 1548-65.

KHANG, M., BINDRA, R.S., AND MARK SALTZMAN, W., 'Intrathecal delivery and its applications in leptomeningeal disease', Adv. Drug Deliv. Rev., 186 (2022 2022).

KHWAJA, J. AND CWYNARSKI, K., 'The Treatment of Primary and Secondary CNS Lymphoma', Clin. Lymphoma Myeloma Leukemia, 23 (2023 2023), S119-S22.

KIM, S., et al., 'High tumoral PD-L1 expression and low PD-1+ or CD8+ tumor-infiltrating lymphocytes are predictive of a poor prognosis in primary diffuse large B-cell lymphoma of the central nervous system', OncoImmunology, 8/9 (2019 2019).

KIM, T.M., et al., 'A Phase 1 Study of ALX148, a CD47 Blocker, in Combination with Rituximab in Patients with Non-Hodgkin Lymphoma', Blood, 134 (2019 2019), 1953.

KIM, S., et al., 'Comparative analysis of clinicopathologic features and tumor immune-microenvironment of primary diffuse large B cell lymphoma of the central nervous system according to molecular classification', Cancer Res., 83/7 (2023 2023).

KIM, S.H., et al., 'The role of local ablative therapy in patients with advanced invasive mucinous adenocarcinoma of the lung', J. Cancer Res. Clin. Oncol., 150/9 (2024 2024).

KLEINSCHMIDT-DEMASTERS, B.K. AND GILANI, A., 'Secondary parenchymal CNS involvement by lymphoma including rare types: Follicular and EBV-positive NK/T cell lymphoma, nasal type', Ann. Diagn. Pathol., 53 (2021 2021).

KOGURE, Y., HASHIMOTO, H., AND OKI, M., 'A Randomized Phase III Study of Pembrolizumab Versus Pembrolizumab-Carboplatin-Pemetrexed for Locally Advanced or Metastatic Nonsquamous Non-small-cell Lung Cancer with PD-L1 50% or more (LAPLACE-50): Study Protocol', Clin. Lung Cancer, 22/6 (2021 2021), e921-e24.

KORFEL, A., 'A focus on pharmacotherapy for primary central nervous system lymphoma', Expert Rev. Hematol., 8/5 (2015 2015), 559-62.

KORFEL, A. AND SCHLEGEL, U., 'Identifying targetable genetic features in primary CNS lymphoma', Int. J. Hematol. Oncol., 5/3 (2016 2016), 93-96.

KORFEL, A., 'Targeted therapy in CNS lymphoma', Oncol. Res. Treat., 39 (2016 2016), 17.

KORFEL, A., et al., 'Case-based review: Primary central nervous system lymphoma', Neuro-Oncol. Pract., 4/1 (2017 2017), 46-59.

KORFEL, A., '"Wonder drugs" in central nervous system lymphoma', Transl. Cancer Res., 6 (2017 2017), S1158-S62.

KOROLEVA, D.A., et al., 'SUCCESSFUL TREATMENT OF RELAPSES OF PRIMARY MEDIASTINAL LARGE B-CELL LYMPHOMA WITH CENTRAL NERVOUS SYSTEM INVOLVEMENT', Gematol. Transfusiologiya, 68/3 (2023 2023), 398-409.

KREIDIEH, F.Y. AND TAWBI, H.A., 'Current and Emerging Options for Patients With Melanoma Brain Metastases', Clin. Adv. Hematol. Oncol., 20/10 (2022 2022), 610-27.

KUHNL, A., et al., 'Real-World Data of High-Grade Lymphoma Patients Treated with CD19 CAR-T in England', Blood, 134 (2019 2019), 767.

KUMAR, A., SAGAR, S., AND KUMAR, K., 'Correspondence to C-reactive protein levels, the prognostic nutritional index, and the lactate dehydrogenase-to-lymphocyte ratio are important prognostic factors in primary central nervous system lymphoma: a single-center study of 223 patients', Neurosurg. Rev., 47/1 (2024 2024).

KVOPKA, M., LAKE, S.R., AND SMITH, J.R., 'Intraocular chemotherapy for vitreoretinal lymphoma: A review', Clin. Exp. Ophthalmol., 48/2 (2020 2020), 240-48.

LACASCE, A., 'Treatment Approach to AYA Patients With Lymphoma', Clin. Lymphoma Myeloma Leukemia, 23 (2023 2023), S8-S10.

LAKSHMANAN, A. AND BYRD, J.C., 'spotlight on ibrutinib in PCNSL: Adding another feather to its cap', Cancer Discov., 7/9 (2017 2017), 940-42.

LANG, F., et al., Molecular biology of EBV in relationship to HIV/AIDS-associated oncogenesis (177; E.S. Robertson, 3610 Hamilton Walk, 201E Johnson Pavilion, Philadelphia, PA, United States, 2019) 81-103.

LAU, L.M.S., et al., 'In vitro and in vivo drug screens of tumor cells identify novel therapies for high-risk child cancer', EMBO Mol. Med., 14/4 (2022 2022).

LAVIE, D., et al., 'Open-Label, Randomized, Phase 3 Study of Coformulated Favezelimab and Pembrolizumab Versus Chemotherapy in Patients with Relapsed or Refractory Classical Hodgkin Lymphoma Refractory to Anti-PD-1 Therapy: Keyform-008', Blood, 142 (2023 2023), 3082.

LEI, M., et al., 'Evaluation of the impact of thyroiditis development in patients receiving immunotherapy with programmed cell death-1 inhibitors', J. Oncol. Pharm. Pract., 25/6 (2019 2019), 1402-11.

LEPIK, K.V., et al., 'Immune checkpoint inhibitor salvage therapy in patient with refractory primary central nervous system lymphoma', Vopr. Onkol., 65/2 (2019 2019), 294-97.

LEWIS, K.L., et al., 'Outcomes for Patients with Primary or Secondary Central System Lymphoma Treated with Ibrutinib: A Multicentre Retrospective Analysis', Blood, 134 (2019 2019), 1620.

LI, X.-D., et al., 'Clinical and correlative analysis of a phase 2 study of lenalidomide and rituximab in previously untreated indolent non-hodgkin lymphoma', Blood, 124/21 (2014 2014).

LI, C., et al., 'Novel CD19-Specific γ/δ TCR-T Cells in Relapsed or Refractory Diffuse Large B-Cell Lymphoma', Blood, 138 (2021 2021), 826.

LI, L., et al., 'Combination of Selinexor with BTK Inhibitor for Central Nervous System Diffuse Large B-Cell Lymphoma, Possible Mechanisms and Therapeutic Potential Exploration', Blood, 142 (2023 2023), 3010.

LI, S., et al., 'Signaling pathways in brain tumors and therapeutic interventions', Signal Transduct. Target. Ther., 8/1 (2023 2023).

LIN, J.J., et al., 'Clinical activity of alectinib in advanced RET-rearranged non-small cell lung cancer', J. Thorac. Oncol., 11/11 (2016 2016), 2027-32.

LIN, N., SONG, Y., AND ZHU, J., 'Immune checkpoint inhibitors in malignant lymphoma: Advances and perspectives', Chin. J. Cancer Res., 32/3 (2020 2020), 303-18.

LIU, Y., YAO, Q., AND ZHANG, F., 'Diagnosis, prognosis and treatment of primary central nervous system lymphoma in the elderly population (Review)', Int. J. Oncol., 58/3 (2021 2021), 371-87.

LIU, B., et al., 'Correlation study of PD-L1, CD4, CD8, and PD-1 in primary diffuse large B-cell lymphoma of the central nervous system', Pathol. Res. Pract., 239 (2022 2022).

LIU, Z., et al., 'ACT001 inhibits primary central nervous system lymphoma tumor growth by enhancing the anti-tumor effect of T cells', Biomed. Pharmacother., 178 (2024 2024).

LOCKE, F.L., et al., 'Zuma-6: Phase 1-2 multicenter study evaluating safety and efficacy of axicabtagene ciloleucel (axi-cel; KTE-C19) in combination with atezolizumab in patients with refractory difiuse large b-cell lymphoma (DLBCL)', J. Clin. Oncol., 35/15 (2017 2017).

LÖW, S. AND BATCHELOR, T.T., 'Primary Central Nervous System Lymphoma', Semin. Neurol., 38/1 (2018 2018), 86-94.

LUKAS, R.V., et al., 'Primary central nervous system lymphoma: Part 2: Modern therapeutic management and future directions', ONCOLOGY, 32/2 (2018 2018), e11-e19.

LUNA, L.P., et al., 'Arterial spin labeling clinical applications for brain tumors and tumor treatment complications: A comprehensive case-based review', Neuroradiol. J., 36/2 (2023 2023), 129-41.

LURAIN, K., et al., 'Radiation-sparing treatment of HIV-related primary central nervous system lymphoma with antiretroviral therapy, rituximab and high-dose methotrexate', Blood, 132 (2018 2018).

M, BARTOLETTI, et al., 'An Exceptional Response to Dostarlimab in Mismatch Repair Deficient, Microsatellite Instability-High and Platinum Refractory Endometrial Cancer', (29; Switzerland2022), 5209-12.

MA, L. AND GONG, Q., 'Recent advances and challenges in primary central nervous system lymphoma: a narrative review', Transl. Cancer Res., 12/5 (2023 2023), 1335-52.

MADABHAVI, I.V., et al., 'Sanctuary site central nervous system relapse-refractory DLBCL responding to nivolumab and lenalidomide', Oral Oncol., 93 (2019 2019), 122-24.

MADDISON, K., et al., 'Low tumour-infiltrating lymphocyte density in primary and recurrent glioblastoma', Oncotarget, 12/21 (2021 2021), 2177-87.

MAJOR, A., et al., 'Efficacy of checkpoint inhibition after CAR-T failure in aggressive B-cell lymphomas: outcomes from 15 US institutions', Blood Adv., 7/16 (2023 2023), 4528-38.

MANOS, K., et al., 'Phase i Dose Escalation Study of Radiotherapy and Durvalumab (MEDI4736) in Relapsed or Refractory Diffuse Large B-cell Lymphoma (DLBCL): The RaDD Study', Cancer Res., 81/13 (2021 2021).

MARCELIS, L., et al., 'In-depth characterization of the tumor microenvironment in central nervous system lymphoma reveals implications for immune-checkpoint therapy', Cancer Immunol. Immunother., 69/9 (2020 2020), 1751-66.

MARCUCCI, F., CORTI, A., AND FERRERI, A.J.M., 'Breaching the blood–brain tumor barrier for tumor therapy', Cancers, 13/10 (2021 2021).

MENGOLI, M.C., et al., 'ALK-positive adenocarcinoma of the lung expressing neuroendocrine markers and presenting as a "pituitary adenoma"', Pathologica, 109/4 (2017 2017), 408-11.

MERRYMAN, R.W., et al., 'Checkpoint blockade in Hodgkin and non-Hodgkin lymphoma', Blood Adv, 1/26 (2017 2017), 2643-54.

MERRYMAN, R.W., et al., 'Immune and cell therapy in non-hodgkin lymphoma', Cancer J., 26/3 (2020 2020), 269-77.

MILES, D., et al., 'IMpassion131: A phase III study comparing 1L atezolizumab with paclitaxel vs placebo with paclitaxel in treatment-naive patients with inoperable locally advanced or metastatic triple negative breast cancer (TNBC)', Cancer Res., 78/4 (2018 2018).

MILLIGAN, M.G., et al., 'Stereotactic Radiation Therapy With Concurrent Immune Checkpoint Inhibitor Therapy in Patients With Relapsed or Refractory Central Nervous System Lymphoma', Int. J. Radiat. Oncol. Biol. Phys., 111/3 (2021 2021), e300-e01.

MINNITI, G., et al., 'Leptomeningeal disease and brain control after postoperative stereotactic radiosurgery with or without immunotherapy for resected brain metastases', J. Immunother. Cancer, 9/12 (2021 2021).

MISYURINA, A., et al., 'Modified r-bfm immunochemotherpy results higher frequency of complete remission than r-daepoch/ r-chop in patients with high-grade b-cell lymphoma double-hit transformed from follicular lymphoma', HemaSphere, 5 (2021 2021), 247-48.

MIYAO, K., YOKOTA, H., AND SAKEMURA, R.L., 'Is CD19-directed chimeric antigen receptor T cell therapy a smart strategy to combat central nervous system lymphoma?', Front. Oncol., 12 (2023 2023).

MIZUSHINA, Y., et al., 'Clinical case of lung spindle cell carcinoma markedly responsive to pembrolizumab', Thorac. Cancer, 12/16 (2021 2021), 2279-82.

MO, S.S., CLEVELAND, J., AND RUBENSTEIN, J.L., 'Primary CNS lymphoma: update on molecular pathogenesis and therapy', Leuk. Lymphoma, 64/1 (2023 2023), 57-65.

MOK, T., et al., 'Updated overall survival and final progression-free survival data for patients with treatment-naive advanced ALK-positive non-small-cell lung cancer in the ALEX study', Annals of Oncology, 31/8 (2020 2020), 1056-64.

MONABATI, A., et al., 'Research paper: Immune checkpoint molecules in primary diffuse large b-cell lymphoma of the central nervous system', Basic Clin. Neurosci., 11/4 (2020 2020), 491-98.

MONDELLO, P., MIAN, M., AND BERTONI, F., 'Primary central nervous system lymphoma: Novel precision therapies', Crit. Rev. Oncol. Hematol., 141 (2019 2019), 139-45.

MONTILLO, M., et al., 'A multicenter, open label, uncontrolled, phase II clinical trial evaluating the safety and efficacy of venetoclax in combination with atezolizumab and obinutuzumab in richter transformation of CLL', J. Clin. Oncol., 37 (2019 2019).

MOSKOP, A., TOWER, R., AND HARKER-MURRAY, P., 'Residual disease detected by pet/mri in cns relapse of primary mediastinal b cell lymphoma', Pediatr. Blood Cancer, 67 (2020 2020).

N, GALLDIKS, P, LOHMANN, AND KJ, LANGEN, Imaging challenges following newer treatment options: are companion diagnostics required in neurooncology? (20; England, 2020) 651-52.

N, GIRAUD, et al., 'Positron-emission tomography-guided radiation therapy: Ongoing projects and future hopes', Cancer radiotherapie : journal de la Societe francaise de radiotherapie oncologique, 24/5 (2020-8 2020), 437-43.

NAKAHARA, Y., et al., 'Intracranial response to nivolumab in a patient with PD-L1-negative lung adenocarcinoma', Intern. Med., 57/21 (2018 2018), 3149-52.

NAYAK, L. AND IWAMOTO, F., 'Treatment of recurrent primary central nervous system lymphoma patients with anti-PD1 antibody nivolumab', Neurology, 86/16 (2016 2016).

NIETO, Y., et al., 'Phase 2 trial of high-dose gemcitabine, busulfan and melphalan with autologous stem cell transplant (ASCT) for patients with refractory hodgkin's lymphoma (HL)', Bone Marrow Transplant., 49 (2014 2014), S166.

NIZAMUDDIN, I., et al., ABCL-607 Trial in Progress: Phase 1 Study With Dose Expansion of Acalabrutinib and Durvalumab (MEDI 4736) in Primary Central Nervous System Lymphoma (24, 2024) S485-S86.

OK, C.Y. AND YOUNG, K.H., 'Targeting the programmed death-1 pathway in lymphoid neoplasms', Cancer Treat. Rev., 54 (2017 2017), 99-109.

OULEE, A., XU, L., AND WORSWICK, S., 'Primary Retroperitoneal Malignant Melanoma with Involvement of Iliac Artery and Vein', Case Rep. Med., 2021 (2021 2021).

PAGES-GELI, C., et al., 'Macrophages Play a Key Role in Controlling Tumor Growth and Response to Immunotherapy in Primary Central Nervous System Lymphoma', Blood, 142 (2023 2023), 1642.

PARK, S.I., et al., 'High-Dose Methotrexate Containing Induction Chemotherapy Followed By Nivolumab Consolidation in Older (≥ 65) Patients with Previously Untreated Primary CNS Lymphoma', Blood, 142 (2023 2023), 1723.

PARKHI, M., et al., 'Prognostic implications of the tumor immune microenvironment and immune checkpoint pathway in primary central nervous system diffuse large B-cell lymphoma in the North Indian population', APMIS, 130/2 (2022 2022), 82-94.

PEIĆ, A.K., COHA, B., AND CANJKO, I., 'CASE REPORT OF SUCCESFUL MULTIDISIPLINARY TREATMENT FOR PATIENT WITH METASTATIC LUNG CANCER AND RELAPSED/REFRACTORY CHRONIC LYMPHATIC LEUKEMIA/SMALL LYMPHOCYTIC LYMPHOMA', Libri Oncol., 51 (2023 2023), 30-31.

PENSATO, U., et al., 'Fulminant cerebral edema following CAR T-cell therapy: case report and pathophysiological insights from literature review', J. Neurol., 269/8 (2022 2022), 4560-63.

PENSATO, U., et al., 'CAR t-cell therapy in BOlogNa–NEUrotoxicity TReatment and Assessment in Lymphoma (CARBON–NEUTRAL): proposed protocol and results from an Italian study', J. Neurol., 270/5 (2023 2023), 2659-73.

PINCHA, R., et al., 'Refractory Primary Mediastinal B-Cell Lymphoma: A Case Report of Conventional Chemotherapies, Immune Checkpoint Inhibitors, Polatuzumab Vedotin, Transplantation, and Post-Transplant Large Granular Lymphocytosis', Indian J. Med. Paediatr. Oncol., 43/6 (2022 2022), 523-27.

PM, ROUSSEAU, et al., 'First case of cutaneous sarcoidosis within tattoos under durvalumab', (58; England2019), e168-e70.

PRIMEAUX, B., et al., 'Characterizing second line and beyond therapies for primary central nervous system lymphomas', Hematol. Oncol., 42/6 (2024 2024).

PRO, S., et al., 'Peripheral nervous system involvement in non-primary pediatric cancer: From neurotoxicity to possible etiologies', J. Clin. Med., 10/14 (2021 2021).

RACHDI, A., et al., 'Recent advances in the diagnosis and the treatment of primary CNS lymphoma', Rev. Neurol., 179/5 (2023 2023), 481-89.

RAMADAN, S., et al., 'Advances in therapeutic strategies for primary CNS B-cell lymphomas', Expert Rev. Hematol., 15/4 (2022 2022), 295-304.

RAMAKRISHNAN, A., et al., 'Phase 1 alexander study of AUTO3 the first bicistronic chimeric antigen receptor (CAR) targeting cd19 and CD22 with pembrolizumab in patients with relapsed/refractory diffuse large b cell lymphoma', HemaSphere, 4 (2020 2020), 80.

RAMIREZ, A., et al., 'NIVOLUMAB-INDUCED CNS DEMYELINATION IN A 15-YEAR-OLD WITH CLASSIC HODGKIN LYMPHOMA', Neuro-Oncology, 25 (2023 2023), i53.

RAMIREZ-GAMERO, A., et al., 'Plasmablastic lymphoma: 2024 update on diagnosis, risk stratification, and management', Am. J. Hematol., 99/8 (2024 2024), 1586-94.

RIBRAG, V., et al., 'An open-label, multicohort Phase Ib trial of pembrolizumab (MK-3475) for advanced hematologic malignancies: KEYNOTE-013', J. Immunother. Cancer, 3 (2015 2015).

ROCHATE, D., et al., 'Autonomic dysfunction as manifestation of ICANS: A case report', Medicine, 103/36 (2024 2024), e38659.

RODDIE, C., et al., 'SAFETY AND EFFICACY FINDINGS OF AUTO1, A FAST-OFF RATE CD19 CAR, IN RELAPSED/REFRACTORY PRIMARY CNS LYMPHOMA', HemaSphere, 6 (2022 2022), 2560-61.

ROSCHEWSKI, M. AND HODSON, D.J., 'Diffuse large B-cell lymphoma involving the central nervous system: biologic rationale for targeted therapy', Haematologica, 109/2 (2024 2024), 388-400.

RÖSLER, W., et al., 'CAR T-cell Infusion Following Checkpoint Inhibition Can Induce Remission in Chemorefractory Post-transplant Lymphoproliferative Disorder of the CNS', HemaSphere, 6/7 (2022 2022).

ROZENBLUM, L., et al., 'Role of Positron Emission Tomography in Primary Central Nervous System Lymphoma', Cancers, 14/17 (2022 2022).

RUBENSTEIN, J.L., 'Biology of CNS lymphoma and the potential of novel agents', Hematology, 2017/1 (2017 2017), 556-64.

---, 'Can rituximab unlock the innate potential of checkpoint blockade in the CNS?', Leuk. Lymphoma, 60/2 (2019 2019), 281-83.

S, CHIA, et al., A Phase Ib Trial of Durvalumab in Combination with Trastuzumab in HER2-Positive Metastatic Breast Cancer (CCTG IND.229) (24; England, 2019) 1439-45.

SALEM, P.A., et al., 'Personalized multimodality therapy (ICT) with immune checkpoint inhibitors (ICI), chemotherapy (CT), and targeted treatment (TT), in advanced/refractory cancer', J. Clin. Oncol., 38/15 (2020 2020).

SAUSEN, D.G., BASITH, A., AND MUQEEMUDDIN, S., 'EBV and Lymphomagenesis', Cancers, 15/7 (2023 2023).

SCHAFF, L.R. AND GROMMES, C., 'Updates on Primary Central Nervous System Lymphoma', Curr. Oncol. Rep., 20/2 (2018 2018).

---, 'Update on novel therapeutics for primary cns lymphoma', Cancers, 13/21 (2021 2021).

SCHAFF, L.R. AND MELLINGHOFF, I.K., 'Glioblastoma and Other Primary Brain Malignancies in Adults: A Review', JAMA, 329/7 (2023 2023), 574-87.

SCHLIFFKE, S., et al., 'Immunophenotyping of tumor-infiltrating t cells in primary CNS lymphoma', Neuro-Oncology, 23 (2021 2021), ii57.

SCHWARTZ, M.S., et al., 'A phase I/II study of blinatumomab in combination with pembrolizumab for adults with relapsed refractory B-lineage acute lymphoblastic leukemia: University of California Hematologic Malignancies Consortium Study 1504', J. Clin. Oncol., 37 (2019 2019).

SETHI, T.K., et al., 'Clinicopathologic correlates of MYD88 L265P mutation and programmed cell death (PD-1) pathway in primary central nervous system lymphoma', Leuk. Lymphoma, 60/12 (2019 2019), 2880-89.

SEVERINSEN, R.S.D., et al., 'Patients with Primary Central Nervous System Lymphoma Have High Levels of Soluble Programmed Cell Death Protein 1 in Their Pretherapeutic Cerebrospinal Fluid', Blood, 138 (2021 2021), 1334.

SHI, H., et al., 'Targeting the tumor microenvironment in primary central nervous system lymphoma: Implications for prognosis', J. Clin. Neurosci., 124 (2024 2024), 36-46.

SHIPP, M.A., 'Gianni bonadonna memorial lecture: “Genetic signatures and targetable pathways in lymphoid malignancies”', Hematol. Oncol., 35 (2017 2017), 23-24.

SHMIDT, D.I., et al., 'Checkpoint inhibitors and conventional therapy for central nervous system lymphoma', Ann. Oncol., 30 (2019 2019), xi21.

SHMIDT, D.I., et al., 'Treatment of patients with central nervous system lymphoma', Cell. Ther. Transplant., 8/3 (2019 2019), 117-18.

SIDAWAY, P., 'Haematological cancer: Nivolumab is effective in PCNSL and PTL', Nat. Rev. Clin. Oncol., (2017 2017).

SIEG, N., et al., 'Treatment patterns and disease course of previously untreated Primary Central Nervous System Lymphoma: Feasibility of MTX-based regimens in clinical routine', Eur. J. Haematol., 107/2 (2021 2021), 202-10.

ŠIMONČIČOVÁ, E., et al., 'Present and future of microglial pharmacology', Trends Pharmacol. Sci., 43/8 (2022 2022), 669-85.

SKADBORG, S., et al., 'Evidence of t cell activation and intratumoral nivolumab-presence in glioblastoma patients treated with nivolumab and bevacizumab', Neuro-Oncology, 23 (2021 2021), vi55.

SUGITA, Y., et al., 'The perivascular microenvironment in Epstein–Barr virus positive primary central nervous system lymphoma: The role of programmed cell death 1 and programmed cell death ligand 1', Neuropathology, 38/2 (2018 2018), 125-34.

SUMRALL, A., et al., 'Frequent high tumor mutational burden (TMB) and PD-L1 expression in primary CNS lymphoma (PCNSL)', Neuro-Oncology, 20 (2018 2018), vi240-vi41.

SUN, R.-F., YU, Q.-Q., AND YOUNG, K.H., 'Critically dysregulated signaling pathways and clinical utility of the pathway biomarkers in lymphoid malignancies', Chronic Dis. Transl. Med., 4/1 (2018 2018), 29-44.

TAI, P., et al., 'Metastatic brain tumors: To treat or not to treat, and with what?', Curr. Cancer Ther. Rev., 16/3 (2020 2020), 168-81.

TAKAKI, M., et al., 'Case of primary cutaneous peripheral T-cell lymphoma, not otherwise specified, with characteristics of follicular helper T cells', J. Dermatol., 41/6 (2014 2014), 529-32.

TAKASHIMA, Y., et al., 'Differential expression of individual transcript variants of PD-1 and PD-L2 genes on Th-1/Th-2 status is guaranteed for prognosis prediction in PCNSL', Sci Rep, 9/1 (2019 2019), 10004.

TAM, C.S., et al., 'An Update on Safety and Preliminary Efficacy of Highly Specific Bruton Tyrosine Kinase (BTK) Inhibitor Zanubrutinib in Combination with PD-1 Inhibitor Tislelizumab in Patients with Previously Treated B-Cell Lymphoid Malignancies', Blood, 134 (2019 2019), 1594.

TAO, K., WANG, X., AND TIAN, X., 'Relapsed Primary Central Nervous System Lymphoma: Current Advances', Front. Oncol., 11 (2021 2021).

TATEISHI, K., et al., 'Primary central nervous system lymphoma: clinicopathological and genomic insights for therapeutic development', Brain Tumor Pathol., 38/3 (2021 2021), 173-82.

TERZIEV, D., et al., 'Autologous re-transplantation and checkpoint inhibitor maintenance in second relapse of primary central nervous system lymphoma', Oncol. Res. Treat., 40 (2017 2017), 72-73.

TÍMÁR, J., 'Biology of KRAS mutations', J. Thorac. Oncol., 12/1 (2017 2017), S138-S39.

TOBIN, J.W.D., et al., 'Pd‐1 and lag‐3 checkpoint blockade: Potential avenues for therapy in b‐cell lymphoma', Cells, 10/5 (2021 2021).

TRIFOI, M., et al., 'SAFETY AND EFFICACY OF INTRA-CSF BIOLOGIC AGENTS IN TREATMENT OF NEOPLASTIC MENINGITIS', Neuro-Oncology, 25 (2023 2023), ii115.

TSANG, M., et al., 'Survival and Patient-Reported Outcomes of Older Adults with Primary Central Nervous System Lymphoma on Low-Dose Lenalidomide', Blood, 136 (2020 2020), 21-22.

TSILIMIDOS, G., et al., 'Successful Rapid Oral Desensitization to Ibrutinib in a Patient With Severe Immediate Hypersensitivity Reaction', Clin. Lymphoma Myeloma Leukemia, 21/10 (2021 2021), e745-e47.

TUOHY, K.J., et al., 'MOLECULAR CHARACTERIZATION OF PRIMARY CENTRAL NERVOUS SYSTEM LYMPHOMA VS NON-CNS LYMPHOMA AND CORRELATION BETWEEN MUTATIONAL PROFILE AND TREATMENT RESPONSE', Neuro-Oncology, 24 (2022 2022), ii59.

VANERWEGEN, E., et al., 'Cerebellar Hypermetabolism in a Hodgkin Lymphoma Leads to Diagnosis of Paucisymptomatic Cryptococcus neoformans Meningitis', Clin. Nucl. Med., 48/8 (2023 2023), E374-E76.

VÁRNAI, C., et al., 'Mortality among Adults with Cancer Undergoing Chemotherapy or Immunotherapy and Infected with COVID-19', JAMA Netw. Open, 5/2 (2022 2022).

VILLASBOAS, J.C., et al., 'The DIAL Study (Dual Immunomodulation in Aggressive Lymphoma): A Randomized Phase 2 Study of CDX-1127 (Varlilumab) in Combination with Nivolumab in Patients with Relapsed or Refractory Aggressive B-Cell Lymphomas (NCI 10089 / NCT03038672)', Blood, 134 (2019 2019), 1591.

VILLASBOAS, J.C., et al., 'The dial study (dual immunomodulation in aggressive lymphoma): Randomized phase 2 trial of varlilumab plus nivolumab in relapsed/refractory aggressive B-cell lymphomas', Hematol. Oncol., 37 (2019 2019), 70-71.

VODICKA, P., KLENER, P., AND TRNENY, M., 'Diffuse Large B-Cell Lymphoma (DLBCL): Early Patient Management and Emerging Treatment Options', OncoTargets Ther., 15 (2022 2022), 1481-501.

VRDOLJAK, E., et al., 'Real-World Safety and Efficacy of Nivolumab in Advanced Squamous and Nonsquamous Non-Small-Cell Lung Cancer: A Retrospective Cohort Study in Croatia, Hungary, and Malta', J. Oncol., 2020 (2020 2020).

WANG, C.-C.J. AND KAPLAN, L.D., 'Clinical management of HIV-Associated hematologic malignancies', Expert Review of Hematology, 9/4 (2016 2016), 361-76.

WANG, Y., et al., 'Clinical Characteristics and Outcome of Diffuse Large B-Cell Lymphoma with 9p24.1 Copy Number Alterations', Blood, 130 (2017 2017).

WANG, Y., et al., 'Amplification of 9p24.1 in diffuse large B-cell lymphoma identifies a unique subset of cases that resemble primary mediastinal large B-cell lymphoma', Blood Cancer J., 9/9 (2019 2019).

WANG, H., et al., 'Immune checkpoint blockade and CAR-T cell therapy in hematologic malignancies', J. Hematol. Oncol., 12/1 (2019 2019).

WANG, L., et al., 'Case report: Successful treatment of a patient with relapsed/refractory primary central nervous system lymphoma with thiotepa-based induction, autologous stem cell transplantation and maintenance', Front. Oncol., 13 (2023 2023).

WANG, W., et al., 'Cell-Free DNA in Cerebrospinal Fluid Complements the Monitoring Value of Interleukin-10 in Newly Diagnosed Primary Central Nervous System Lymphoma', J. Oncol., 2023 (2023 2023).

WANG, Y., et al., 'Serological response and immune-related adverse events following COVID-19 vaccination in cancer patients treated with immune checkpoint inhibitors: A systematic review and meta-analysis', Rev. Med. Virol., 34/1 (2024 2024).

WESTIN, J.R., et al., 'A Phase II Trial of Nivolumab and Ibrutinib for Patients with Relapsed or Refractory Central Nervous System Lymphoma', Blood, 134 (2019 2019), 4086.

WESTIN, J., et al., 'Nivolumab and Ibrutinib for Treatment of Patients with Refractory or Relapsed Central Nervous System Lymphoma', Blood, 142 (2023 2023), 1721.

WHITE, M., et al., 'MYD88 L265P mutation and CDKN2A loss as early mutational events in primary central nervous system lymphomas', J. Clin. Oncol., 36/15 (2018 2018).

WIRSCHING, H.-G., et al., 'Targeted therapies and immune checkpoint inhibitors in primary cns lymphoma', Cancers, 13/12 (2021 2021).

Y, WU, et al., 'Anlotinib combined with durvalumab in a patient with recurrent multifocal brain metastases of small cell lung cancer after definitive concurrent chemoradiotherapy and palliative radiotherapy of the lung and brain: a case report', (10; China2021), 2379-86.

YAMSHON, S., et al., 'Safety and Toxicity Profiles of CAR T Cell Therapy in Non-Hodgkin Lymphoma: A Systematic Review and Meta-Analysis', Clin. Lymphoma Myeloma Leukemia, 24/6 (2024 2024), e235-e56.e2.

YANG, H., et al., 'Advances and challenges in the treatment of primary central nervous system lymphoma', J. Cell. Physiol., 235/12 (2020 2020), 9143-65.

YARCHOAN, R., et al., 'Malignancies in People with HIV: Successes and Challenges at the Intersection of Virology, Immunology, and Oncology', Cancer Res., 83/7 (2023 2023).

YING, Z., et al., 'A phase II study of anti-PD-1 sintilimab in combination with chidamide and azacitidine in refractory and relapsed peripheral T-cell lymphoma', Hematol. Oncol., 39 (2021 2021), 347-48.

YOU, H., BALUSZEK, S., AND KAMINSKA, B., 'Immune microenvironment of brain metastases—Are microglia and other brain macrophages little helpers?', Front. Immunol., 10 (2019 2019).

YOU, H., et al., 'Genomic complexity is associated with epigenetic regulator mutations and poor prognosis in diffuse large B-cell lymphoma', OncoImmunology, 10/1 (2021 2021).

YU, W., et al., 'Real-world experience of commercial relmacabtagene autoleucel (relma-cel) for relapsed/refractory central nervous system lymphoma: a multicenter retrospective analysis of patients in China', J. Immunother. Cancer, 12/5 (2024 2024).

YUAN, Y., et al., 'Current and emerging therapies for primary central nervous system lymphoma', Biomarker Res., 9/1 (2021 2021).

ZAFFIRI, L. AND CHAMBERS, E.T., 'Screening and Management of PTLD', Transplantation, 107/11 (2023 2023), 2316-28.

ZENG, Z., et al., 'NcRNAs: Multi-angle participation in the regulation of glioma chemotherapy resistance (Review)', Int. J. Oncol., 60/6 (2022 2022).

ZENG, Z., et al., 'A Phase II Study of the Efficacy and Safety of the Srmt (Sintilimab, Rituximab, Methotrexate, and Temozolomide) Regimen in Newly Diagnosed Primary Central Nervous System Lymphoma', Blood, 142 (2023 2023), 857.

ZENG, Z., et al., 'Sintilimab (anti-PD-1 antibody) combined with high-dose methotrexate, temozolomide, and rituximab (anti-CD20 antibody) in primary central nervous system lymphoma: a phase 2 study', Signal Transduct. Target. Ther., 9/1 (2024 2024).

ZHAI, Y., ZHOU, X., AND WANG, X., 'Novel insights into the biomarkers and therapies for primary central nervous system lymphoma', Ther. Adv. Med. Oncol., 14 (2022 2022).

ZHANG, R., et al., 'Improved Safety and Efficacy of a Multi-Target Chimeric Antigen Receptor Modified T Cell Therapy (4SCAR2.0) Against Relapsed or Refractory Lymphomas', Blood, 136 (2020 2020), 47.

ZHANG, W., et al., 'Case report: CD19-directed CAR-T cell therapy combined with BTK inhibitor and PD-1 antibody against secondary central nervous system lymphoma', Front. Immunol., 13 (2022 2022).

ZHANG, X., et al., 'A Phase I/II Study of Orelabrutinib Combined with Anti-Programmed Cell Death Protein-1 Antibody and Fotemustine for Patients with Newly Diagnosed Primary Central Nervous System Lymphoma (PCNSL)', Blood, 140 (2022 2022), 12075-76.

ZHANG, Y., et al., 'PRELIMINARY RESULTS OF A PHASE II STUDY OF ORELABRUTINIB IN COMBINATION WITH ANTI-PD-1 MONOCLONAL ANTIBODY IN REFRACTORY OR RELAPSED PRIMARY CNS LYMPHOMA', HemaSphere, 6 (2022 2022), 256-57.

ZHANG, A.S., et al., 'Endoscopic Endonasal Biopsy for Diagnosis of Undifferentiated Lesions of the Cavernous Sinus', World Neurosurg., 175 (2023 2023), e391-e96.

ZHOU, X.A., et al., 'Genomic landscape of primary cutaneous diffuse large B cell lymphoma, leg type', Blood, 130 (2017 2017).

ZHOU, Y. AND XU, X., 'Application of new targeted drugs in relapsed/refractory primary central nervous system lymphoma', Hematology, 27/1 (2022 2022), 105-12.

ZHOU, Y., et al., 'Sustained response following BTK inhibitors based treatment in HIV-related primary central nervous system lymphoma: case report', AIDS Res. Ther., 20/1 (2023 2023).

ZHUANG, L., et al., 'Third time’s a charm? Mobilization of autologous peripheral blood stem cells in patients with two previous failed mobilizations with plerixafor', Transfusion, 60/6 (2020 2020), 1253-59.

ZOROFCHIAN, S., et al., 'Characterization of genomic alterations in primary central nervous system lymphomas', J. Neuro-Oncol., 140/3 (2018 2018), 509-17.

ZOU, R., et al., 'Long-term Complete Remission of Decitabine-Primed Tandem CD19/CD22 CAR-T Therapy with PD-1 and BTK Inhibitors Maintenance in a Refractory Primary Central Nervous System Lymphoma Patient', Cancer Res. Treat., 55/4 (2023 2023), 1363-68.

**Excluded due to wrong treatment/ incorrect phase of treatment**

TERZIEV, D., et al., 'Nivolumab maintenance after salvage autologous stem cell transplantation results in long-term remission in multiple relapsed primary CNS lymphoma', Eur. J. Haematol., 101/1 (2018 2018), 115-18.

CAO, S., et al., 'A 65-Year-Old Male with Primary Central Nervous System Diffuse Large B-Cell Lymphoma on Nivolumab with Oral Mucositis and Targetoid Plaques', Dermatopathology, 4/1 (2017 2017), 13-17.

FENG, C., et al., 'NEW-ONSET DIABETIC KETOACIDOSIS SECONDARY TO NIVOLUMAB THERAPY IN A PATIENT WITH PRIMARY CENTRAL NERVOUS SYSTEM LYMPHOMA', Osteopath. Fam. Phys., 13/3 (2021 2021), 40-43.

GULLICKSON, C., et al., 'Isolated central nervous system relapse in two adolescents with primary mediastinal large B-cell lymphoma after treatment with R-DA-EPOCH', Pediatr. Blood Cancer, 71/8 (2024 2024).

CHIAPPELLA, A., et al., 'Checkpoint Inhibition before Axicabtagene Ciloleucel Cell Therapy in Primary Mediastinal B-Cell Lymphoma (PMBCL) Treated in Real Life Setting', Blood, 136 (2020 2020), 12-13.

**Excluded due to insufficient participant**

GRABER, J.J., et al., 'Pembrolizumab immunotherapy for relapsed CNS Lymphoma', Leuk. Lymphoma, 61/7 (2020 2020), 1766-68.

**Excluded due to incorrect disease/ stages of the disease**

JOFFE, E., et al., 'A phase I and randomized phase II etctn study of KW-0761 (Mogamulizumab) and MK-3475 (Pembrolizumab) in relapsed and refractory diffuse large B-cell lymphoma', J. Clin. Oncol., 38/15 (2020 2020).

JEONG, A.-R., et al., 'Higher tumor mutational burden and PD-L1 expression correlate with shorter survival in hematologic malignancies', Ther. Adv. Med. Oncol., 16 (2024 2024).

**Included**

Chukwueke U, Wright I, Muzikansky A, Russ A, Fontana B, Kats V, et al. CTIM-39. A PHASE 1B STUDY OF PEMBROLIZUMAB, IBRUTINIB AND RITUXIMAB IN RECURRENT/REFRACTORY (RR) PRIMARY CENTRAL NERVOUS SYSTEM LYMPHOMA (PCNSL). Neuro-Oncology. 2023;25(Supplement_5):v71-v2. doi: 10.1093/neuonc/noad179.0279.

Gavrilenko AN, Volkov NP, Shmidt DI, Polushin AY, Kondakova E, Lepik KV, et al. Nivolumab in Primary CNS Lymphoma and Primary Testicular Lymphoma with CNS Involvement: Single Center Experience. Blood. 2020;136(Supplement 1):4-. doi: 10.1182/blood-2020-138924.

Gavrilenko A, Markelov V, Volkov N, Shmidt D, Skiba Y, Kondakova E, et al. PB2308: NIVOLUMAB-BASED THERAPY OF RELAPSED OR REFRACTORY PRIMARY LARGE B-CELL LYMPHOMA OF IMMUNE-PRIVILEGED SITES AND DLBCL WITH SECONDARY CNS INVOLVEMENT. Hemasphere. 2023;7(Suppl). Epub 20230808. doi: 10.1097/01.HS9.0000975956.49127.b4. PubMed Central PMCID: PMCPMC10429729.

Hoang-Xuan K, Houot R, Soussain C, Blonski M, Schmitt A, Delwail V, et al. First Results of the Acsé Pembrolizumab Phase II in the Primary CNS Lymphoma (PCNSL) Cohort. Blood. 2020;136(Supplement 1):15-6. doi: 10.1182/blood-2020-141773.

Nayak L, Iwamoto FM, LaCasce A, Mukundan S, Roemer MGM, Chapuy B, et al. PD-1 blockade with nivolumab in relapsed/refractory primary central nervous system and testicular lymphoma. Blood. 2017;129(23):3071-3. Epub 20170329. doi: 10.1182/blood-2017-01-764209. PubMed PMID: 28356247; PubMed Central PMCID: PMCPMC5766844.

Westin J, Nair R, Fayad L, Iyer SP, Malpica L, Neelapu SS, et al. Nivolumab and Ibrutinib for Treatment of Patients with Refractory or Relapsed Central Nervous System Lymphoma. Blood. 2023;142(Supplement 1):1721-. doi: 10.1182/blood-2023-191123.

Yi JH, Kim SJ, Kim SA, Jung J, Yoon DH. Nivolumab in Relapsed or Refractory Primary CNS Lymphoma: Multicenter, Retrospective Study. Blood. 2023;142(Supplement 1):1772-. doi: 10.1182/blood-2023-186554.
